# Protecting the confidentiality and security of personal health information in low- and middle-income countries in the era of SDGs and Big Data

**DOI:** 10.3402/gha.v9.32089

**Published:** 2016-11-23

**Authors:** Eduard J. Beck, Wayne Gill, Paul R. De Lay

**Affiliations:** 1 UNAIDS LAC Regional Support Team, Georgetown, Guyana; 2 Balsillie School of International Affairs, Ontario, ON, Canada; 3 Global Health Advisor, Washington, DC, USA

**Keywords:** personal health information, privacy laws, confidentiality, security, stigma, discrimination, ethics, SDGs, Big Data

## Abstract

**Background:**

As increasing amounts of personal information are being collected through a plethora of electronic modalities by statutory and non-statutory organizations, ensuring the confidentiality and security of such information has become a major issue globally. While the use of many of these media can be beneficial to individuals or populations, they can also be open to abuse by individuals or statutory and non-statutory organizations. Recent examples include collection of personal information by national security systems and the development of national programs like the Chinese Social Credit System. In many low- and middle-income countries, an increasing amount of personal health information is being collected. The collection of personal health information is necessary, in order to develop longitudinal medical records and to monitor and evaluate the use, cost, outcome, and impact of health services at facility, sub-national, and national levels. However, if personal health information is not held confidentially and securely, individuals with communicable or non-communicable diseases (NCDs) may be reluctant to use preventive or therapeutic health services, due to fear of being stigmatized or discriminated against. While policymakers and other stakeholders in these countries recognize the need to develop and implement policies for protecting the privacy, confidentiality and security of personal health information, to date few of these countries have developed, let alone implemented, coherent policies. The global HIV response continues to emphasize the importance of collecting HIV-health information, recently re-iterated by the *Fast Track to End AIDS by 2030* program and the recent changes in the *Guidelines on When to Start Antiretroviral Therapy and on Pre-exposure Prophylaxis for HIV*. The success of developing HIV treatment cascades in low- and middle-income countries will require the development of National Health Identification Systems. The success of programs like Universal Health Coverage, under the recently ratified Sustainable Development Goals is also contingent on the availability of personal health information for communicable and non-communicable diseases.

**Design:**

Guidance for countries to develop and implement their own guidelines for protecting HIV-information formed the basis of identifying a number of fundamental principles, governing the areas of privacy, confidentiality and security. The use of individual-level data must balance maximizing the benefits from their most effective and fullest use, and minimizing harm resulting from their malicious or inadvertent release.

**Discussion:**

These general principles are described in this paper, as along with a bibliography referring to more detailed technical information. A country assessment tool and user's manual, based on these principles, have been developed to support countries to assess the privacy, confidentiality, and security of personal health information at facility, data warehouse/repository, and national levels. The successful development and implementation of national guidance will require strong collaboration at local, regional, and national levels, and this is a pre-condition for the successful implementation of a range of national and global programs.

**Conclusion:**

This paper is a call for action for stakeholders in low- and middle-income countries to develop and implement such coherent policies and provides fundamental principles governing the areas of privacy, confidentiality, and security of personal health information being collected in low- and middle-income countries.

## Introduction

UNAIDS recently announced that 15 million people living with HIV (PLHIV) were on anti-retroviral therapy (ART) (1). As part of scaling up HIV services in low- and middle-income countries, increasing amount of personal health information is being collected (2). The need to further scale up therapeutic services is one consequence from the evolving guidelines that now recommend starting ART when a PLHIV is diagnosed, irrespective of CD4 count (3–6). Currently, an estimated 36.9 million (range: 34.3–41.4 million) people are living with HIV across the world (1); hence, many countries will need to scale up both HIV therapeutic and prevention services. The need for increased provision of preventive and therapeutic services will result in increased collection of individual-level health information, especially given the recent UNAIDS *Fast Track to End AIDS by 2030* treatment targets, including ‘90-90-90’ and ‘95-95-95’ (7).

The life-expectancy of PLHIV has increased considerably over the past decade, largely due to increased access to ART. Even in low- and middle-income countries, life-expectancy is now comparable with that for people not living with HIV (8–10). Over time, a considerable proportion of PLHIV are likely to develop non-HIV-related comorbidities, mainly non-communicable diseases (NCDs), which will require them to seek treatment and care from NCDs service providers. In addition, for many populations in low- and middle-income countries, NCDs have become important causes of morbidity and mortality and these patients will also require access to prevention and therapeutic services. The new emphasis by the World Health Organization and the United Nations Sustainable Development Goals (SDGs) (11) for *Universal Health Coverage* (12) and NCDs (13), respectively, will require improved management of patient data, including confidentiality and security of those data.

Individual-level information needs to be collected every time a person uses therapeutic or prevention health services. The primary reason to collect information is to optimize patient management and build accurate and longitudinal medical records to document the results of investigations, interventions, and changes in health status, as a consequence of the use of these services. A secondary reason is to use pseudo-anonymized or de-identified personal health information to monitor and evaluate the use, cost, outcome, and impact of health services at facility, sub-national, and national levels.

While it is widely recognized that PLHIV in many countries are stigmatized and discriminated against (14), either because they are living with HIV or because they are part of a marginalized population (15), similar stigma and discrimination is also observed against people with other communicable diseases (16) or NCDs (17, 18). Many countries therefore need to confront the issue of keeping patient information confidential and secure, while ensuring appropriate access to such data to improve services.

The UNESCO *Universal Declaration on Bioethics and Human Rights*, Article 9, states: ‘*The privacy of the persons concerned and the confidentiality of their personal information should be respected. To the greatest extent possible, such information should not be used or disclosed for purposes other than those for which it was collected or consented to, consistent with international law, in particular international human rights law*’ (19).

Health data should be used to improve health and reduce harm (20). This must be a continuous process that balances the benefits that can be derived from fully accessing the data, while trying to reduce any harm that can result from either the accidental or deliberate release of individually identifiable data (20–22).

In 2006, UNAIDS and PEPFAR organized a workshop in Geneva, Switzerland, to discuss and develop guidance for countries to enable them to develop and implement their own guidelines for protecting HIV information. The participants included clinicians, public health physicians, bio-ethicists, lawyers, informatics experts, community members, and PLHIV from across the various regions. Topics varied from privacy and human rights to technical aspects of how to secure paper- and electronic-based information. These activities were supported by an extensive literature review available to all participants. The outcome of the workshop was summarized in the report, *Interim Guidelines on Protecting the Confidentiality and Security of HIV Information* (20).

As a follow-up to the workshop and publication of the *Interim Guidelines*, a questionnaire was developed and sent to UNAIDS field staff covering 98 low- and middle-income countries. The aim of this exercise was to assess whether low- and middle-income countries, which were scaling up HIV services, had developed and implemented guidelines to protect the confidentiality and security of HIV information (23). Results indicated that few countries had actually developed guidelines covering the areas of *privacy*, *confidentiality*, and *security*. Based on the responses from countries, it was clear that many informants had not understood the relationship between these three concepts. For example, of the 49 countries claiming to have developed privacy laws, 55% reported that they had not developed any guidelines for the implementation of such laws. Of those countries that reportedly had developed policy guidelines, their implementation lacked the breadth and depth of those set out in the *Interim Guidelines* (23).

Further follow-up to the workshop also included the development of an assessment tool. The tool assesses the level of protection of personal health information at facility, data warehouse/repository, and national levels. Although the assessment tool was based on the *Interim Guidelines*, it went beyond a singular focus on HIV information. It was felt that all principles and items covered in the *Interim Guidelines* were applicable to the confidentiality and security of all personal health information.

The first drafts of the assessment tool were developed by professionals from Macro International Inc.^®^ (Atlanta) working with colleagues from CDC Atlanta and UNAIDS. These drafts were extensively reviewed, and eventually, a draft was discussed and reviewed by participants at a workshop held in Lusaka, Zambia, in 2012 (24). This workshop again had multi-stakeholder participation, including members of the Ministry of Health (MOH), clinicians, members of civil society, PLHIVs, and staff from UNAIDS, CDC, and others.

## The assessment tool

Recommendations from the Zambia workshop and other relevant inputs were reviewed in 2013. These recommendations, together with inputs from the *Data Security and Confidentiality Guidelines for HIV, Viral Hepatitis, Sexually Transmitted Disease, and Tuberculosis Programs* (25), formed the basis of a draft of the assessment tool. Survey questions were agreed for health facility, data warehouse, and national levels, and the assessment tool was completed in June 2014.

The assessment tool was field-tested in Jamaica, in September 2014. The focus of the field-testing was to determine suitability of the assessment tool and its questions in each of the three modules including that for health facilities, data warehouses, and national policy. For each level, a set of questions are contained within a number of major headings, each of which have a number of sub-headings (Box 1). Jamaica was selected as the field-test country because it has an established, well-developed health sector, is English speaking, and has a population size that was considered appropriate for the assessment tool to be field-tested.

***Box 1*. Structural outline of the assessment tool (35)**

For each of the three modules, a set of questions are comprised under the following major headings:

- Governance and Policy
- Data Collection (not included at policy level)
- Data Storage
- Data Backup (not included at policy level)
- Authorization and Access Control
- Data Release
- Transmission Security
- Data Disposal

The major headings include a series of subheadings and relevant questions as can be seen in the example of Governance and Policy below:

- Policy
- Governance Structure
- Review of Security Practices
- Responsibilities and Training
- Monitoring Security Breaches
- Conducting Risk Assessments
- Connectivity to Other Networks

Based on discussions with relevant members of the MOH, the tool was field-tested in two primary, two secondary, and two tertiary care facilities. In addition, the tool was also field-tested at a national data warehouse and at the national policy level. A work-plan was developed, based on discussions between relevant members of MOH, CDC and UNAIDS, other government officials, health and legal professionals, and members of civil society that also agreed to participate in the process.

The primary method of data collection was through small group interviews, reaching consensus on each question of the assessment tool. Additionally, if policies or procedures existed in that department a copy of the guidelines was requested. The field-test documented responses to the questions by identifying the existing national policies, legislation, technical guidelines, including scope and coverage, for the sole purpose of assessing the questions on the tool and its ability to capture the data of interest.

In general, constructive comments and suggestions were made regarding changes to the assessment tool. Although most of the questions were easy to understand, they elicited rich discussions, which did increase the length of the meetings. This enabled consensus to be reached on each question. In most instances, the discussion eventually focused on how the assessment tool should be rolled out, implemented, or moved forward in terms of informing policies and procedures. Only 18 questions, out of a total of 168 questions in the tool, needed to be modified or clarified. The field-test confirmed that the assessment tool had been well understood by informants and produced useful information.

The approach used in selecting and vetting the questions was considered to have been thorough based on the feedback session held at the end of the field-test. The scope of the assessment tool was considered to have adequately covered all facets of confidentiality and security and dealt with all relevant aspects of the *Interim Guidelines on Protecting the Confidentiality and Security of HIV Information*. However, the assessment tool is a general tool and does not need major adaptation to be useful in country.

## Privacy, confidentiality, and security principles

The activities described above have led to the identification of a number of fundamental principles, governing the areas of privacy, confidentiality, and security (Box 2). These principles deal with the entire process – from the collection of individual-level information, their storage, use for individual service provision, transmission and dissemination within countries, program monitoring or evaluation, and use by international organizations. They comprise the foundation on which operational recommendations should be based.

***Box 2*. Principles of protecting personal health information (20)**

1. The purpose of defining personal health information confidentiality and security principles is to ensure that health data are available and used to serve the improvement of health, as well as the reduction of harm, for all people, healthy and not healthy. Pursuing this goal involves an ongoing process of refining the balance between maximizing of benefits, which can and should come from the wise and fullest use of data, and protection from harm, which can result from either malicious or inadvertent inappropriate release of individually identifiable data. Appropriate policies, procedures, and technical methods must be balanced to protect both individual and public rights.
2. Personal health information is generally obtained at the point of care, where services are delivered to individuals. Personal identifiable information is individual-level information that includes personal identifiers such as names and addresses. They are managed at community and health facilities whether sponsored by the public sector, NGOs, the private sector, or international organizations. However, in some cases such data are stored in regional or national databases. This category of data also includes national identification numbers, which can be directly linked to individual patients across different databases across various social sectors, for example, the social security number in the United States.
3. The public health goal is to safeguard the health of communities through the collection, analysis, dissemination, and use of health data, which must be carefully balanced with the individual's right to privacy and confidentiality. Guidelines must allow for consideration of relevant cultural norms, which may influence these policies, while ethical principles should guide decision-making regarding the appropriate use and dissemination of data. Overall, guiding principles should be based on human rights principles.
4. For protecting personal health information, three interrelated concepts have an impact on the development and implementation of protections for sensitive data. These are privacy, confidentiality, and security. Privacy is both a legal and an ethical concept. The legal concept refers to the legal protection that has been accorded to an individual to control both access to and use of personal information and provides the overall framework within which both confidentiality and security are implemented. Confidentiality relates to the right of individuals to protection of their data during storage, transfer, and use, in order to prevent unauthorized disclosure of that information to third parties. Security is a collection of technical approaches that address issues covering physical, electronic, and procedural aspects of protecting information collected as part of the scale-up of HIV services.
5. The risk of harm following a breach of confidentiality varies with the national or local context according to levels of stigma, lack of comprehensive public health safety nets, legal traditions of respect of privacy, religious perspectives, and other local conditions. Within countries, privacy and confidentiality laws should be in place, or developed if not already in place, and relevant parameters of privacy or confidentiality laws must be reviewed and known by those involved with the data at all administrative levels. The greatest threats to electronic information systems are generally not from outside attack, but rather from issues inherent in the system design and implementation. These threats fall into two categories: non-availability of data due to system failure and user errors.
6. Development and review of confidentiality and security laws and procedures should include active participation from relevant stakeholders, including civil society and community members, healthcare professionals, information technology specialists, and legal and ethical experts.
7. Funding organizations should comply with these standards and have an obligation to make adequate funding available to implement them, sufficient to ensure protection of the data collected and used. Funding organization must also require that maintaining these standards is a condition for funding of any implementing partners or agencies.
8. A number of organizational procedures need to be followed to ensure safeguards for the collection, transfer, storage, use, dissemination, and disposal of personal identified data and other information. Policies and procedures developed must cover both paper-based and electronic systems. Countries and organizations at all levels of the healthcare system should have a written policy that defines security procedures concerning the way data are collected, stored, transferred, and released. The policies need to be implemented at all relevant levels, and staff must understand the policies and to have signed an agreement stating that they will implement them as part of their work. This will also require training new staff and updating all staff on the relevant procedures.
9. Data may be shared between organizations provided that the data are used for legitimate health purposes. The confidentiality and security measures of the organization receiving the data need to be equivalent to those that collected the data.

## Operational recommendations

These principles give rise to a number of operational recommendations. It is critical that written security procedures be produced, defining how data should be collected, stored, transferred, and released. These written policies should be accessible, understood, and implemented at all organizational and administrative levels. Staff members need to confirm, in writing, that they have read and understood the policies and that they will adhere to them (20).

Individuals who are authorized to access personal health information should also receive appropriate training, at regular intervals. Security strategies and related laws and policies should be continuously reviewed, independently assessed, and revised when required (20).

Cross-sectional or longitudinal paper-based or electronic information, collected as part of clinical management, need to be stored in locked cabinets or locked computers, respectively, within a locked room and a secured building. Paper-based information should be transported in secure briefcases, transmitted by secured fax or using protected mail services within or between organizations (20). Geographically dispersed electronic infrastructures, such as wide area networks, need to be safeguarded via domain encryption and passwords, or other authentication schemas (20).

Security breaches and loss of confidentiality require investigation. Organizations and individuals who are unable to adequately protect the confidentiality and security of identifiable information should be held accountable and appropriate penalties imposed. Improvements then need to be implemented (20). Confidentiality and Security Officers should be identified by organizations at all levels of the national healthcare system and by international organizations.

It is important to delineate potential limits and restrictions on access to personal data. For example, individual-level information should generally not be shared with law enforcement, immigration control, management of the public welfare system, or other non-health functions without consent from the individual to whom the information relates, except in circumstances involving the threat of imminent danger of grave physical harm to individuals or populations (20). The development and implementation of such policies and procedures should be integrated into national strategic health plans. This will become more important as an increasing number of countries are collecting and using personal information for a large variety of social uses, either through their national surveillance systems (26) or through specifically designed programs, such as the Social Credit System currently under development in China. In this system, personal information on ‘… behaviour will be integrated into one comprehensive assessment of you as a person, which will then be used to make you eligible or ineligible for certain jobs, or social services’ (27, 28).

These are just some of the operational issues associated with maintaining the confidentiality and security of personal health information. For detailed information on some of the specific issues surrounding these and other related technical issues, the extended bibliography (Supplementary file) can refer the reader to more detailed technical guidance.

## Call to action

If the personal identifiable health information is not kept confidential and secure, under-pinned by relevant privacy laws, people with communicable or non-communicable diseases may be stigmatized and discriminated against, hampering therapeutic and preventive health measures at individual and population levels (29). Furthermore, if these data are not held securely, maintaining their confidentiality, people may be unwilling to be tested or link up with services due to fears of stigma and discrimination (14, 30). High HIV-related stigma has been found to be directly associated with low social support, poor physical and mental health, and inversely associated with age and income (31).

The collection of personal health information will also enable more detailed information to be available for monitoring and evaluating the use, cost, outcome, and impact of services at facility, sub-national, and national levels (32). Systems to protect this data should be designed to ensure patient confidentiality, but, at the same time, allow relatively easy access to the information at both individual and aggregate levels (20). System availability, including the identification and management of predictable risks to data systems, like electricity interruptions, staffing shortages, or natural disasters, also need to be addressed (20).

However, there continues to be a general lack of interest and progressive action in this area. So far, few countries have systematically developed, let alone, implemented such policies. Why is this occurring?

Possible reasons for this general lack of progress may be due to a variety of reasons. These may include cultural reasons where the local culture does not recognize the need for individual privacy or confidentiality of personal information, the relative lack of development of the country's healthcare and health information systems, perceived financial costs of the development and implementation of such guidelines, and possible disagreements as to which statutory or non-statutory organization ought to have access to such data. However, if countries do not currently invest in their health information systems as part of developing their healthcare system, the long-term social and financial costs are likely to exceed the necessary short-term investment (33).

The development of healthcare systems may provide an opportunity for countries to develop and implement such guidelines. For instance, countries in Asia, sub-Saharan Africa, and the Caribbean are currently in the process of developing unique healthcare identifiers. Such identifiers should, however, be developed and implemented in parallel with national guidelines on the privacy, confidentiality, and security of personal health information (34).

To assist countries in this process, an UNAIDS/PEPFAR assessment tool and user manual have been finalized and are available in paper-format in English (35, 36), while French and Spanish translations are forthcoming. This tool should enable countries to assess the confidentiality and security of personal health information at facility, data warehouse/repository, and national levels, including the review of national policies and privacy laws (35). An electronic version of the tool is currently under development. Additional developments of the tool could include a section assessing the views and understanding of privacy, confidentiality, and security held by patients using the healthcare system of that country.

Personal information is increasingly being collected and used by local, national, and global statutory and non-statutory organizations as part of the broader utilization of Big Data (37). The successful implementation of programs like the *Fast Track to End AIDS by 2030* (7), implementing Universal Health Coverage (11) and the monitoring of the SDGs (12) and the general improvements in healthcare in many countries, is contingent on the successful protection of the confidentiality and security of personal health information, guaranteed by the implementation of national policies and privacy laws. To achieve this, strong collaboration among healthcare professionals, information technology specialists, ethicists, academics, civil society representatives, and policy-makers at local, sub-national, national and global levels is required.

## Supplementary Material

Protecting the confidentiality and security of personal health information in low- and middle-income countries in the era of SDGs and Big Data

## Paper context

This paper is a call for action for stakeholders in low- and middle-income countries and global organizations, to develop and implement coherent policies on protecting the confidentiality and security of personal health information. At a time of increased demand for personal health information, required for the success of national and global health services and programs, it provides fundamental principles governing the areas of privacy, confidentiality and security of personal health information as well as referring to more detailed technical information.
