# Supplementary material for: Protecting the confidentiality and security of personal health information in low- and middle-income countries in the era of SDGs and Big Data
Source: Glob Health Action. 2016 Nov 23;9:10.3402/gha.v9.32089. doi: 10.3402/gha.v9.32089 (PMC5123209; doi:10.3402/gha.v9.32089)
Supplement: Protecting the confidentiality and security of personal health information in low- and middle-income countries in the era of SDGs and Big Data [file GHA-9-32089-s001.pdf]

Appendix 1: Protecting the Privacy, Confidentiality and Security of Personally Identifiable Health Information: extended bibliography

This bibliography accompanies the paper *Protecting the Privacy, Confidentiality and Security of Personally Identifiable Health Information* and was updated up to September 2014.

The key databases used to search new material included: ACM Digital Library, CDC, EBSCOhost, Federal Information Processing Standards Publication Series of the National Institute of Standards and Technology (NIST), Google Scholar, IEEE Xplore Digital Library, Mendeley, National Center for Biotechnology Information (NCBI), New York University School of Law, North American Association of Central Cancer Registries, PubMed, Research Gate, Science Direct, Source National Archives (UK), Standards and Technology (NIST), The Academy of Medical Sciences, The Cochrane Library and the Wiley On Line Library.

The keywords used to search new material were as follows:

Personally identifiable health data; information security policy; rights and access to information; protecting patient privacy; data confidentiality; threat and vulnerability identification; computer security; encryption health information; firewalls; transmission of health data; informed consent; unique health identifiers; public key infrastructure; disclosure health information; paper archives; storage policy; data release policy; biometric readers; smartcards; and probabilistic matching.

1. Recommended Readings

Overall

1. Bath PA. Health informatics: **"Current Issues and Challenges,"** J Inf Sci. 2008;34:501-518. *doi:10.1177/0165551508092267.* (To purchase this article please use the following link: http://jis.sagepub.com/content/34/4/501)

   Health informatics concerns the use of information and information and communication technologies within healthcare. Health informatics and information science need to take account of the unique aspects of health and medicine. The development of information systems and electronic records within health needs to consider the information needs and behaviour of all users. The sensitivity of personal health data raises ethical concerns for developing electronic records. E-health initiatives must actively involve users in the design, development, implementation and evaluation, and information science can contribute to understanding the needs and behaviour of user groups. Health informatics could make an important contribution to the ageing society and to reducing the digital divide and health divides within society. There is a need for an appropriate evidence base within health informatics to support future developments, and to ensure health informatics reaches its potential to improve the health and well-being of patients and the public.

2. Beck EJ, Mandalia S, Harling G, Santas XM, Mosure D, Delay PR. "**Protecting HIV information in countries scaling up HIV services: a baseline study,"** *J Int AIDS Soc*. 2011;14:6. doi:10.1186/1758-2652-14-6. http://archive.biomedcentral.com/1758-2652/14/6

Individual-level data are needed to optimize clinical care and monitor and evaluate HIV services. Confidentiality and security of such data must be safeguarded to avoid stigmatization and discrimination of people living with HIV. We set out to assess the extent that countries scaling up HIV services have developed and implemented guidelines to protect the confidentiality and security of HIV information.

3. Blakemore M, Craglia M. **"Access to Public-Sector Information in Europe: Policy, Rights, and Obligations,"** *Inf Soc*. 2006;22:13-24. doi:10.1080/01972240500388180. (To purchase this article please use the following link: http://www.tandfonline.com/doi/abs/10.1080/01972240500388180?journalCode=utis20#.U9uvsvJDslM)

The article reviews the debates and policies on access to public- sector information (PSI) in Europe in relation to the contests between policies of open access, rights of access to PSI by citizens and business, and the assessment of the cost benefits of PSI to the economy and society. The political dimension of these debates within the European Union is highlighted to demonstrate the complexities of the governance of information within a pan-European regulatory framework.

4. Center for Disease Control (CDC). "**Data Security and Confidentiality Guidelines for HIV, Viral Hepatitis, Sexually Transmitted Disease, and Tuberculosis Programs,"** Atlanta; 2011. Available at: http://www.cdc.gov/nchhstp/programintegration/docs/PCSIDataSecurityGuidelines.pdf.

This document recommends standards for all NCHHSTP programs that, when adopted, will facilitate the secure collection, storage, and use of data while maintaining confidentiality. Designed to support the most desirable practices for enabling secure use of surveillance data for public health action and ensuring implementation of comprehensive evidence- based prevention services, the standards are based on 10 guiding principles that provide the foundation for the collection, storage, and use of these public health data. They address five areas: program policies and responsibilities, data collection and use, data sharing and release, physical security, and electronic data security. Intended for use by state and local health department disease programs to inform the development of policies and procedures, the standards are intentionally broad to allow for differences in public health activities and response across disease programs.

5. Chetley, A., Davies, J., Trude, B., McConnell, H., & Ramirez, R. (2006). **"Improving health connecting people: the role of ICTs in the health sector of developing countries,"** (7), 58. Retrieved from http://www.asksource.info/pdf/framework.pdf

The paper describes the major constraints and challenges faced in using information and communications technology (ICT) effectively in the health sector of developing countries. It draws out good practice for using ICT in the health sector, identifies major players and stakeholders and highlights priority needs and issues of relevance to policy makers.

6. Crawford K, Schultz J. Big Data and Due Process: **"Toward A Framework to Redress Predictive Privacy Harms,"** *Bost Coll Law Rev Vol 55, No 1, 2014*. 2013:1-31. http://www.media-alliance.org/downloads/SSRN-id2325784.pdf

The rise of "big data" analytics in the private sector poses new challenges for privacy advocates. Unlike previous computational models that exploit personally identifiable information (PII) directly, such as behavioral targeting, big data has exploded the definition of PII to make many more sources of data personally identifiable. By analyzing primarily metadata, such as a set of predictive or aggregated findings without displaying or distributing the originating data, big data approaches often operate outside of current privacy protections (Rubinstein 2013; Tene and Polonetsky 2012), effectively marginalizing regulatory schema. Big data presents substantial privacy concerns – risks of bias or discrimination based on the inappropriate generation of personal data – a risk we call "predictive privacy harm."

Predictive analysis and categorization can pose a genuine threat to individuals, especially when it is performed without their knowledge or consent. While not necessarily a harm that falls within the conventional "invasion of privacy" boundaries, such harms still center on an individual's relationship with data about her. Big data approaches need not rely on having a person's PII directly: a combination of techniques from social network analysis, interpreting online behaviors and predictive modeling can create a detailed, intimate picture with a high degree of accuracy. Furthermore, harms can still result when such techniques are done poorly, rendering an inaccurate picture that nonetheless is used to impact on a person's life and livelihood.

7. Cucoranu IC, Parwani A V, West AJ, et al. **"Privacy and security of patient data in the pathology laboratory,"** *J Pathol Inform*. 2013;4:4. doi:10.4103/2153-3539.108542. http://www.pubmedcentral.nih.gov/articlerender.fcgi?artid=3624703&tool=pmcentrez&rendertype=abstract

Data protection and security are critical components of routine pathology practice because laboratories are legally required to securely store and transmit electronic patient data. With increasing connectivity of information systems, laboratory work-stations, and instruments themselves to the Internet, the demand to continuously protect and secure laboratory information can become a daunting task. This review addresses informatics security issues in the pathology laboratory related to passwords, biometric devices, data encryption, internet security, virtual private networks, firewalls, anti-viral software, and emergency security situations, as well as the potential impact that newer technologies such as mobile devices have on the privacy and security of electronic protected health information (ePHI). In the United States, the Health Insurance Portability and Accountability Act (HIPAA) govern the privacy and protection of medical information and health records. The HIPAA security standards final rule mandate administrative, physical, and technical safeguards to ensure the confidentiality, integrity, and security of ePHI. Importantly, security failures often lead to privacy breaches, invoking the HIPAA privacy rule as well. Therefore, this review also highlights key aspects of HIPAA and its impact on the pathology laboratory in the United States.

8. Dolgin E. **"New data protection rules could harm research, science groups say,"** *Nat Med*. 2014;20(3):224. (To purchase this article please use the following link: http://dx.doi.org/10.1038/nm0314-224b)

Unrest is stirring in Europe over a proposed amendment to the EU's draft General Data Protection Regulation that would prohibit researchers from using individual medical records for research unless explicit consent for that purpose has been given by patients. The policy, if implemented, would dramatically reduce the ability to conduct investigations involving data from disease registries and stymie cohort studies, which obtained more general consent from their participants years ago.

9. eHealth Ontario. ***"Guide to Information Security for the Health Care Sector,"*** 2010. Available at: http://www.ehealthontario.on.ca/images/uploads/pages/documents/InfoSecGuide_Complex.pdf.

The privacy and security of information is of prime importance to all individuals, government agencies and private sector organizations. Nowhere is the protection of information a more sensitive issue than in the health care sector. Like many other industries, health care is becoming more efficient in delivering clinical results and more cost effective through the use of Information Technology (IT), including computers, applications, electronic networks and related technologies. However, the use of these technologies and the increasing exchange of health information among health providers also pose a privacy and security risk to personal information (PI) and personal health information (PHI). Health information that is disclosed to unauthorized individuals, accessed incorrectly, tampered with, or lost could result in devastating impacts on patient health or even life. In 2007 the Ontario Health Informatics

Standards Council (OHISC) approved the development of an information security guide based on the internationally recognized standards ISO 17799:20051 and ISO 27001:2005, as Ontario's minimum requirements to support the implementation of the province's eHealth vision. This guide focuses on two priorities: • Building an information security program • Setting up a risk management program

10. Hammond WE, Bailey C, Boucher P, Spohr M, Whitaker P. **"Connecting Information To Improve Health,"** *Health Aff*. 2010;29:284-288. doi:10.1377/hlthaff.2009.0903. http://content.healthaffairs.org/content/29/2/284

Effective health information systems require timely access to all health data from all sources, including sites of direct care. In most parts of the world today, these data most likely come from many different and unconnected systems--but must be organized into a composite whole. We use the word interoperability to capture what is required to accomplish this goal. We discuss five priority areas for achieving interoperability in health care applications (patient identifier, semantic interoperability, data interchange standards, core data sets, and data quality), and we contrast differences in developing and developed countries. Important next steps for health policy makers are to define a vision, develop a strategy, identify leadership, assign responsibilities, and harness resources.

11. Beck, E. J., Mandalia, S., Harling, G., Santas, X. M., Mosure, D., & Delay, P. R. (2011). Protecting HIV information in countries scaling up HIV services: a baseline study. *Journal of the International AIDS Society*, *14*, 6. doi:10.1186/1758-2652-14-6

CDC. (2011). *Data Security and Confidentiality Guidelines for HIV, Viral Hepatitis, Sexually Transmitted Disease, and Tuberculosis Programs*. Atlanta. Retrieved from http://www.cdc.gov/nchhstp/programintegration/docs/PCSIDataSecurityGuidelines.pdf

UNAIDS/PEPFAR. (2007). *Interim Guidelines on Protecting the Confidentiality and Security of HIV Information* (pp. 1–62). Retrieved from

http://data.unaids.org/pub/manual/2007/confidentiality_security_interim_guidelines_15may2007_en.pdf

The NAACCR data exchange record layouts were designed to facilitate electronic transmission of cancer registry data among registries for multiple purposes. The layouts can be used to provide standardized data from reporting sources to central registries; to share tumor reports on residents of other states/provinces from one central registry to another; or to report data from diverse facilities or states/provinces contributing to a combined study. The NAACCR data set is comprised of all data items recommended for use by the major cancer registry standard-setting organizations.

12. Health Informatics. **"Health Informatics: Identification of Subjects of Health Care,"** Geneva; 2011. (To purchase use the following link: http://www.iso.org/iso/home/store/catalogue_tc/catalogue_detail.htm?csnumber=59755)

This technical specification identifies the data elements and relevant structure and content of the data used to manually identify individuals in a health-care setting and provides support to the identification of individuals in a consistent manner between systems that will support the natural changes in usage and application of the various names used by people over time. This document addresses the business requirements of identification and the data needed to improve the confidence of health service providers and subjects of care identification. It defines the data used to identify subjects of care and the business processes associated with this activity, whether computerized or manual. This document is intended to be used to support both identification of subjects of care by individuals and computerized identification in automated

matching systems.

13. Health IT. **"Privacy & Security Policy,"** 2013. Available at: http://www.healthit.gov/policy-researchers-implementers/privacy-security-policy.

Health information technology promises a number of potential benefits for individuals, health care providers, and the nation's health care system. It has the ability to advance clinical care, improve population health, and reduce costs. At the same time, this environment also poses new challenges and opportunities for protecting individually identifiable health information. This website unfolds the federal policies and regulations that are in place to help protect patient privacy and guide the nation's adoption of health information technology.

14. Hofferkamp J. **"Standards for Cancer Registries Volume III: Standards for Completeness, Quality, Analysis, Management, Security and Confidentiality of Data,"** 2008 http://www.naaccr.org/LinkClick.aspx?fileticket=hvFzJKUcRM8%3D&tabid=134&mid=474.

The Procedure Guidelines for Cancer Registries being developed by the ROC focuses on individual operational activities at the central registry level. The intent is to supplement Volume III by providing detailed guidelines for specific operations activities.

15. *Jean-Baptiste R, Gebhard I.* **"Series IV: Cancer Case Ascertainment: Procedure Guidelines for Cancer Registries,"** *2002 https://www.naaccr.org/LinkClick.aspx?fileticket=fsZdXjDtP78%3D&tabid=130&mid=470.*

As a North American standard-setting body for central population-based cancer registries, NAACCR recognizes the existence of several issues that impact registries' ability to achieve uniformity in cancer registration. This document reflects the results of a consensus exercise to examine best practices in registries in the United States and Canada. This work has been conducted under the auspices of the NAACCR Registry Operations Committee as part of the NAACCR initiative to document best practices for registries.

16. Klein WT, Havener LA. **"North American Association of Central Standards for Cancer Registries Volume V Pathology Laboratory Electronic Reporting,"** 2011. http://www.naaccr.org/LinkClick.aspx?fileticket=KA9zQRZ5Vn8%3D&tabid=136&mid=476

The scope of this document is limited to standards and guidelines to transmit cancer information from pathology laboratories to cancer registries. The standard format documents address data items, data item definitions, and transmission specifications. Implementation guidelines and business rules are incorporated to help cancer registries, pathology laboratories, and vendors within North America respond to the call for cancer cases in a uniform method. In addition, the use of HL7 as the primary recommended clinical data interchange standard will provide a cost-effective solution to addressing data exchange in the 21st century.

17. Martin E, Helbig N, Shah N. **"Liberating Data to Transform Health Care: New York's Open Data Experience,"** *JAMA*. 2014;311(24). (To purchase this article please use the following link: http://jama.jamanetwork.com/article.aspx?articleid=1883027)

The health community relies on governmental survey, surveillance, and administrative data to track epidemiologic trends, identify risk factors, and study the health care delivery system. Since 2009, a quiet "open data" revolution has occurred. Catalyzed by President Obama's open government directive, federal, state, and local governments are releasing de-identified data meeting 4 "open" criteria: public accessibility, availability in multiple formats, free of charge, and unlimited use and distribution rights.1 As of February 2014, HealthData.gov, the

federal health data repository, has more than 1000 data sets, and Health Data NY, New York's health data site, has 48 data sets with supporting charts and maps. Data range from health interview surveys to administrative transactions. The implicit logic is that making governmental data readily available will improve government transparency; increase opportunities for research, mobile health application development, and data-driven quality improvement; and make health-related information more accessible. Together, these activities have the potential to improve health care quality, reduce costs, facilitate population health planning and monitoring, and empower health care consumers to make better choices and live healthier lives.

18. McGuire AL, Beskow LM. **"Informed consent in genomics and genetic research,"** *Annu Rev Genomics Hum Genet*. 2010;11:361-381. doi:10.1146/annurev-genom-082509-141711. http://www.ncbi.nlm.nih.gov/pmc/articles/PMC3216676/pdf/nihms331692.pdf

There are several features of genetic and genomic research that challenge established norms of informed consent. In this paper, we discuss these challenges, explore specific elements of informed consent for genetic and genomic research conducted in the United States, and consider alternative consent models that have been proposed. All of these models attempt to balance the obligation to respect and protect research participants with the larger social interest in advancing beneficial research as quickly as possible.

19. Molina AD, Salajegheh M, Fu K. HICCUPS : **"Health Information Collaborative Collection Using Privacy and Security Categories and Subject Descriptors,"** *ACM - SPIMACS*. 2009:21-30. doi:10.1145/1655084.1655089. http://www.cs.jhu.edu/~sdoshi/jhuisi650/discussion/hiccups.pdf

A recent national survey suggests that the HIPAA privacy rule has not only failed to preserve patient privacy adequately, but also has had a negative impact on clinical research. Our work suggests that researchers revisit the possibilities of homomorphic encryption and apply the techniques to secure aggregation of medical telemetry. A primary goal is to maintain the privacy of individual patient records while also allowing clinical researchers to have flexible access to aggregated information. We discuss the preliminary design of HICCUPS, a distributed system that uses homomorphic encryption to allow only the caregivers to have unrestricted access to patients' records and at the same time enable researchers to compute statistical values and aggregation functions across different patients and caregivers. In the context of processing medical telemetry, we advocate expressibility of aggregation functions more than fast computation as a primary metric of system quality. Copyright 2009 ACM.

20. Prada SI, Gonzalez-Martinez C, Borton J, et al. **"Avoiding Disclosure of Individually Identifiable Health Information: A Literature Review,"** *SAGE Open*. 2011;1. doi:10.1177/2158244011431279. http://sgo.sagepub.com/content/early/2011/12/12/2158244011431279.full-text.pdf+html

Achieving data and information dissemination without harming anyone is a central task of any entity in charge of collecting data. In this article, the authors examine the literature on data and statistical confidentiality. Rather than comparing the theoretical properties of specific methods, they emphasize the main themes that emerge from the ongoing discussion among scientists regarding how best to achieve the appropriate balance between data protection, data utility, and data dissemination. They cover the literature on de-identification and reidentification methods with emphasis on health care data. The authors also discuss the benefits and limitations for the most common access methods. Although there is abundant theoretical and empirical research, their review reveals lack of consensus on fundamental questions for empirical practice: How to assess disclosure risk, how to choose among disclosure methods, how to assess reidentification risk, and how to measure utility loss.

21. Rodrigues Joel, de la Torre Isabel, Fernández Gonzalo L-CM. **"Analysis of the Security and Privacy Requirements of Cloud-Based Electronic Health Records Systems,"** *J od Med Internet Res*. 2013;15(8). Available at:

http://www.ncbi.nlm.nih.gov/pmc/articles/PMC3757992/?report=classic.

The Cloud Computing paradigm offers eHealth systems the opportunity to enhance the features and functionality that they offer. However, moving patients' medical information to the Cloud implies several risks in terms of the security and privacy of sensitive health records. To protect the confidentiality of patient information and facilitate the process, some suggestions for health care providers are made. Moreover, security issues that Cloud service providers should address in their platforms should be considered.

22. Silva BM, Rodrigues JJPC, Canelo F, Lopes IC, Zhou L. **"A data encryption solution for mobile health apps in cooperation environments,"** *J Med Internet Res*. 2013;15:e66. doi:10.2196/jmir.2498. http://www.pubmedcentral.nih.gov/articlerender.fcgi?artid=3636327&tool=pmcentre

Mobile Health (mHealth) proposes health care delivering anytime and anywhere. It aims to answer several emerging problems in health services, including the increasing number of chronic diseases, high costs on national health services, and the need to provide direct access to health services, regardless of time and place. mHealth systems include the use of mobile devices and apps that interact with patients and caretakers. However, mobile devices present several constraints, such as processor, energy, and storage resource limitations. The constant mobility and often-required Internet connectivity also exposes and compromises the privacy and confidentiality of health information. This paper presents a proposal, construction, performance evaluation, and validation of a data encryption solution for mobile health apps.

23. Salido J, Manager SP, Group TC, Corporation M, Cavit D. **"A Guide to Data Governance for Privacy , Confidentiality , and Compliance,"** *Microsoft Trust Computer*. 2010;Part:35. Available at: https://privacyassociation.org/media/pdf/knowledge_center/Guide_to_Data_Governance_Part5_Moving_to_Cloud_Computing_whitepaper.pdf.

The past decade has seen an unprecedented accumulation of data. Organizations in general and business models in particular increasingly rely on confidential data such as intellectual property, market intelligence, and customers' personal information. Maintaining the privacy and confidentiality of this data, as well as meeting the requirements of a growing list of related compliance obligations, are top concerns for government organizations and enterprises alike. Looking ahead to the coming decade, we can see that with cloud computing, organizations will increasingly have to address the challenges of data protection and compliance. This will require implementing a cross-disciplinary effort within the organization—involving human resources, information technology (IT), legal, and other groups—to devise solutions that address privacy and confidentiality in a holistic way. Data governance is one such approach.

24. Taitsman JK, Grimm CM, Agrawal S. **"Protecting Patient Privacy and Data Security,"** *N Engl J Med*. 2013;368:977-979. doi:10.1056/NEJMp1215258. http://www.nejm.org/doi/pdf/10.1056/NEJMp1215258

On December 4, 2012, two Australian radio DJs called London's King Edward VII's Hospital, identified themselves, in fake British accents, as Queen Elizabeth and Prince Charles, and asked about a celebrity patient who had been admitted for pregnancy complications. A nurse, filling in at the reception desk in the early morning hours, answered the phone and, without attempting to verify the callers' identities, transferred them to the duty nurse caring for the Duchess of Cambridge. The duty nurse then provided them with confidential patient information.1 The Australian DJs broadcast the phone call, considering it a humorous prank, but as the world knows, it had disastrous consequences.

25. Beck, E. J., Mandalia, S., Harling, G., Santas, X. M., Mosure, D., & Delay, P. R. (2011). Protecting HIV information in countries scaling up HIV services: a baseline study. *Journal of the International AIDS Society*, *14*, 6. doi:10.1186/1758-2652-14-6

CDC. (2011). *Data Security and Confidentiality Guidelines for HIV, Viral Hepatitis, Sexually*

Transmitted Disease, and Tuberculosis Programs. Atlanta. Retrieved from http://www.cdc.gov/nchhstp/programintegration/docs/PCSIDataSecurityGuidelines.pdf

UNAIDS/CDC. (2007). *Interim Guidelines on Protecting the Confidentiality and Security of HIV Information* (pp. 1–62). Retrieved from http://data.unaids.org/pub/manual/2007/confidentiality_security_interim_guidelines_15may2007_en.pdf

26. The Information for Development Program (infoDev) **"Improving health, connecting people: the role of Information and Communication Technologies in the health sector of developing countries,"** 2006, 58 p. http://www.asksource.info/pdf/framework.pdf

The paper describes the major constraints and challenges faced in using information and communications technology (ICT) effectively in the health sector of developing countries. It draws out good practice for using ICT in the health sector, identifies major players and stakeholders and highlights priority needs and issues of relevance to policy makers.

27. UK Department of Health. **"*The Caldicott Guardian Manual 2010,*"** London; 2010. Available at: http://webarchive.nationalarchives.gov.uk/20130107105354/http://www.dh.gov.uk/prod_consum_dh/groups/dh_digitalassets/@dh/@en/@ps/documents/digitalasset/dh_114506.pdf.

The manual, which is a DH publication, is guidance that takes account of developments in information management in the NHS & in Councils with Social Care responsibilities since the publication of the Caldicott report. It sets out the role of the Caldicott Guardian within an organizational Caldicott/confidentiality function as a part of broader Information Governance.

28. Verhulst S, Noveck BS, Caplan R, Brown K, Paz C. **" The open data era in Health and Social Care,"** 2014. Available at: http://images.thegovlab.org/wordpress/wp-content/uploads/2014/06/nhs-full-report.pdf.

The United Kingdom has been a leader in the open data movement – a new movement by governments around the world to open up the vast repositories of data they hold across agencies and departments, and to collect new kinds of data for public use. Open data is publicly available data that can be universally and readily accessed, used, and redistributed free of charge. It is changing the way governments, nonprofits, and the private sector use data to understand public issues and solve problems in areas as diverse as financial regulation, energy, education, and more. Open data holds particular potential in the health sector. By releasing health data to patients and, when appropriate, on an anonymized basis to researchers and the public, governments and healthcare organizations are betting on the power of greater openness of data to improve the quality of care, lower healthcare costs, and facilitate patient choice. The NHS has made and continues to make significant investments in opening data. Over the past several years, it has launched a series of initiatives that have already had a positive impact on patient education, healthcare choice, healthcare costs, and patient outcomes. Now the NHS is planning a broader, more ambitious programme that has the potential to serve as a worldwide model for the opening of data in healthcare. The purpose of this report is to help design this programme, establishing priorities and ways of measuring impact to guide this and future efforts at data transparency.

29. Wjst M. **"Caught you: threats to confidentiality due to the public release of large-scale genetic data sets,"** *BMC Med Ethics*. 2010;11:21. doi:10.1186/1472-6939-11-21. http://www.ncbi.nlm.nih.gov/pubmed/21190545

Large-scale genetic data sets are frequently shared with other research groups and even released on the Internet to allow for secondary analysis. Study participants are usually not informed about such data sharing because data sets are assumed to be anonymous after stripping off personal identifiers.

30. Yee G, Korba L, Song R. **"Ensuring privacy for E-health services,"** In: *Proceedings - First International Conference on Availability, Reliability and Security, ARES 2006*.Vol 2006.; 2006:321-328. doi:10.1109/ARES.2006.59. http://www.researchgate.net/publication/4238719_Ensuring_privacy_for_e-health_services

The growth of the Internet has been accompanied by the growth of e-health services (e.g. online medical advice, online pharmacies). This proliferation of services and the increasing regulatory and legal requirements for personal privacy have fueled the need to protect the personal privacy of service users. Existing approaches for privacy protection such as access control are predicated on the e-service provider having possession and control over the user's personal data. In this paper, we propose a new approach to protecting personal privacy for e-health services: keeping possession and control over the user's personally identifiable information in the hands of the user as much as possible. Our approach can also be characterized as distributing personally identifiable information only on a "need to know" basis.

Legal, Ethical

31. Adjerid I, Padman R. **"Impact of health disclosure laws on health information exchanges,"** *AMIA Annu Symp Proc*. 2011;2011:48-56. Available at: http://www.pubmedcentral.nih.gov/articlerender.fcgi?artid=3243116&tool=pmcentrez&rendertype=abstract.

Health information exchanges (HIEs) are expected to facilitate data sharing between healthcare entities, thereby improving the efficiency and quality of care. Privacy concerns have been consistently cited as one of the primary challenges to HIE formation and success. Currently, it is unclear how privacy laws - in particular, legislation restricting the disclosure of health records - have shaped the development of HIEs. This preliminary study explores the landscape of state-level health privacy legislation and examines the impact of variations in such privacy and confidentiality laws on the progress of HIEs. We found that states with stronger privacy laws, limiting the disclosure of health information, had significantly more HIEs exchanging data and had fewer failed HIEs. We suggest that this counterintuitive finding may be explained by the more subtle benefits of such laws, such as increased confidence and trust of participants in an exchange.

32. Amy L. Fairchild, Lance Gable, Lawrence O. Gostin, Ronald Bayer, Patricia Sweeney, Robert S. Janssen **"Public Goods, Private Data: HIV and the History, Ethics, and Uses of Identifiable Public Health Information,"** 2007 Public Health Reports 2007 Supplement 1, Vol. 122

A concise history and discussion on the use of personal identifiable information for public health purposes.

33. Brown C. **"Health-care data protection and biometric authentication policies: comparative culture and technology acceptance in China and in the United States,"** *Rev Policy Res*. 2012. Available at: http://onlinelibrary.wiley.com/doi/10.1111/j.1541-1338.2011.00546.x/abstract.

A proliferation of health information technology policies to implement dimensions of e-health, including electronic medical records, electronic health records, personal health records and e-prescribing – along with expanding initiatives on mobile health in developed countries and emerging technologies – has sparked academic inquiry into the protection of privacy and data and the technology to protect privacy and data. This article examines health information technology policies in the United States and in China and the use of authentication technologies to assess biometrics as privacy's friend or foe in different political frameworks with varying conceptions of privacy. An analysis of privacy in the context of health data protection, challenging relations of trust between patients and providers, the increasing perspective of health data integrity as a cyber-security issue, and the growing rate of medical fraud and medical identity theft may yield findings of a convergence of views of privacy and biometrics unexpected of contrasting political cultures.

34. CDC **"HIPAA privacy rule and public health, guidance from CDC and the U.S. DHHS,"** 2003, 24 p.
http://www.cdc.gov/mmwr/pdf/other/m2e411.pdf

The U.S. DHHS issued the Privacy Rule to implement the requirement of the Health Insurance Portability and Accountability Act of 1996 (HIPAA). The purpose of this report is to help public health agencies and others understand and interpret their responsibilities under the HIPAA Privacy Rule. Elsewhere, comprehensive DHHS guidance is located at the HIPAA website of the Office for Civil Rights.

35. Cockcroft S, Sandhu N, Norris A. **"How does national culture affect citizens' rights of access to personal health information and informed consent?,"** *Health Informatics J*. 2009;15:229-243. doi:10.1177/1460458209337444.
http://jhi.sagepub.com/content/15/3/229.full.pdf

Two widely discussed and debated aspects of health law literature are 'informed' consent to medical treatment and the right of access to personal health information. Both are tied to the larger subject of patients' rights, including the right to privacy. This article looks at the issue of informed consent internationally, and goes further to explain some of the inequalities across the world with respect to informed consent and patients' rights legislation via an analysis of the take-up of key legislative attributes in patient consent. Specifically, the effect that national culture, as defined by the GLOBE variables, has on the rate and pattern of adoption of these consent elements is analysed using binary logistic regression to provide evidence of the existence or otherwise of a cultural predicate of the legislative approach. The article concludes by outlining the challenges presented by these differences.

36. Cohen S. **"Nudging and informed consent,"** *Am J Bioeth*. 2013;13:3-11. doi:10.1080/15265161.2013.781704. (To purchase this article please use the following link: http://www.ncbi.nlm.nih.gov/pubmed/23641835)

Libertarian paternalism's notion of "nudging" refers to steering individual decision making so as to make choosers better off without breaching their free choice. If successful, this may offer an ideal synthesis between the duty to respect patient autonomy and that of beneficence, which at times favours paternalistic influence. A growing body of literature attempts to assess the merits of nudging in health care. However, this literature deals almost exclusively with health policy, while the question of the potential benefit of nudging for the practice of informed consent has escaped systematic analysis. This article focuses on this question. While it concedes that nudging could amount to improper exploitation of cognitive weaknesses, it defends the practice of nudging in a wide range of other conditions. The conclusion is that, when ethically legitimate, nudging offers an important new paradigm for informed consent, with a special potential to overcome the classical dilemma between paternalistic beneficence and respect for autonomy.

37. DLA Piper's. **"Data Protection Laws of the World,"** 2014. Available at:
http://www.dlapiperdataprotection.com/#handbook/world-map-section

More than ever it is crucial that organisations manage and safeguard personal information and address their risks and legal responsibilities in relation to processing personal data, to ensure consistency with the growing thicket of applicable data protection legislation. This handbook sets out an overview of the key privacy and data protection laws and regulations across 72 different jurisdictions, and offers a primer to businesses as they consider this complex area of compliance.

38. Gostin LO, Hodge JG, Valdiserri RO **"Informational privacy and the public's health: the Model State Public Health Privacy Act,"** Am J Public Health. 2001;91(9):1388-92. http://www.pubmedcentral.nih.gov/articlerender.fcgi?artid=1446788&tool=pmcentrez&rendertype=abstract.

Protecting public health requires the acquisition, use, and storage of extensive health-related information about individuals. The electronic accumulation and exchange of personal data promises significant public health benefits but also threatens individual privacy; breaches of privacy can lead to individual discrimination in employment, insurance, and government programs. Individuals concerned about privacy invasions may avoid clinical or public health tests, treatments, or research. Although individual privacy protections are critical, comprehensive federal privacy protections do not adequately protect public health data, and existing state privacy laws are inconsistent and fragmented. The Model State Public Health Privacy Act provides strong privacy safeguards for public health data while preserving the ability of state and local public health departments to act for the common good.

39. Kim SYH, Miller FG. **"Informed Consent for Pragmatic Trials - The Integrated Consent Model,"** *N Engl J Med*. 2014;370:769-772. doi:10.1056/NEJMhle1312508. (To purchase this article please use the following link: http://www.ncbi.nlm.nih.gov/pubmed/24552326)

The authors argue that informed consent is ethically necessary in pragmatic trials that randomly assign individual patients to treatments, even when treatment options are within the standard of care. They propose integration of a streamlined consent process into routine practice.

40. Knoppers BM, Harris JR, Budin-Ljøsne I, Dove ES. **"A human rights approach to an international code of conduct for genomic and clinical data sharing,"** *Hum Genet*. 2014;133(7):895-903. doi:10.1007/s00439-014-1432-6. http://www.pubmedcentral.nih.gov/articlerender.fcgi?artid=4053599&tool=pmcentrez&rendertype=abstract

Fostering data sharing is a scientific and ethical imperative. Health gains can be achieved more comprehensively and quickly by combining large, information-rich datasets from across conventionally silo-ed disciplines and geographic areas. While collaboration for data sharing is increasingly embraced by policymakers and the international biomedical community, we lack a common ethical and legal framework to connect regulators, funders, consortia, and research projects so as to facilitate genomic and clinical data linkage, global science collaboration, and responsible research conduct. Governance tools can be used to responsibly steer the sharing of data for proper stewardship of research discovery, genomics research resources, and their clinical applications. In this article, we propose that an international code of conduct be designed to enable global genomic and clinical data sharing for biomedical research. To give this proposed code universal application and accountability, however, we propose to position it within a human rights framework. This proposition is not without precedent: international treaties have long recognized that everyone has a right to the benefits of scientific progress and its applications, and a right to the protection of the moral and material interests resulting from scientific productions. It is time to apply these twin rights to internationally collaborative genomic and clinical data sharing.

41. Lawrence O. Gostin, Zita Lazzarini, Kathleen M. Flaherty **"Legislative Survey of State Confidentiality Laws, with Specific Emphasis on HIV and Immunization,"**

http://www.epic.org/privacy/medical/cdc_survey.html

Examines current U.S. state and federal laws protecting the confidentiality of health information. It focuses on four specific areas: public health information held by government, privately held health care information, HIV and AIDS-related information, and immunization information.

42. Lawrence O. Gostin **"Public Health Law and Ethics: A Reader,"** 2003 http://www.publichealthlaw.net/reader/index.html

Provides a discussion and analysis of critical problems at the interface of law, ethics, and public health. It is intended as a stand-alone text and offers a detailed commentary that defines a public health problem in each chapter, frames the relevant questions, and introduces the selected readings. The commentary also provides additional resources, many of which are included on the web site, for readers interested in further pursuing the subject matter in the chapter. See especially chapter 7 "Public health and the protection of individual rights" and chapter 10 "Surveillance and public health research: privacy and the 'right to know.'"

43. Malin B, Karp D, Scheuermann R. **"Technical and policy approaches to balancing patient privacy and data sharing in clinical and translational research,"** *Clin Res*. 2010;58(1):11-18. doi:10.231/JIM.0b013e3181c9b2ea.Technical. http://www.ncbi.nlm.nih.gov/pmc/articles/PMC2836827/pdf/nihms172884.pdf

Clinical researchers need to share data to support scientific validation and information reuse, and to comply with a host of regulations and directives from funders. Various organizations are constructing informatics resources in the form of centralized databases to ensure widespread availability of data derived from sponsored research. The widespread use of such open databases is contingent on the protection of patient privacy. In this paper, we review several aspects of the privacy-related problems associated with data sharing for clinical research from technical and policy perspectives. We begin with a review of existing policies for secondary data sharing and privacy requirements in the context of data derived from research and clinical settings. In particular, we focus on policies specified by the U.S. National Institutes of Health and the Health Insurance Portability and Accountability Act and touch upon how these policies are related to current, as well as future, use of data stored in public database archives. Next, we address aspects of data privacy and "identifiability" from a more technical perspective, and review how biomedical databanks can be exploited and seemingly anonymous records can be "reidentified" using various resources without compromising or hacking into secure computer systems. We highlight which data features specified in clinical research data models are potentially vulnerable or exploitable. In the process, we recount a recent privacy-related concern associated with the publication of aggregate statistics from pooled genome-wide association studies that has had a significant impact on the data sharing policies of NIH-sponsored databanks. Finally, we conclude with a list of recommendations that cover various technical, legal, and policy mechanisms that open clinical databases can adopt to strengthen data privacy protections as they move toward wider deployment and adoption.

44. Neurodiagn J. **"Ethical considerations in internet use of electronic protected health information,"** *PubMed*. 2012;52:34-41. Available at: http://www.ncbi.nlm.nih.gov/pubmed/22558645.

Caregivers, patients, and their family members are increasingly reliant on social network websites for storing, communicating, and referencing medical information. The Health Insurance Portability and Accountability Act (HIPAA) Privacy Rule seeks balance by protecting the privacy of patients' health information and assuring that this information is available to those who need it to provide health care. Though federal and state governments have created laws and policies to safeguard patient privacy and confidentiality, the laws are inadequate against the rapid and innovative use of electronic health websites. As Internet use broadens access to information, health professionals must be aware that this information is not

always secure. We must identify and reflect on medical ethics issues and be accountable for maintaining privacy for the patient.

45. Office of the Privacy Commissioner of Canada. **"Legal information related to PIPEDA,"** 2013. Available at: https://www.priv.gc.ca/leg_c/interpretations_02_e.asp.

One of the Commissioner's primary roles is to investigate and try to resolve privacy complaints against organizations. Her findings on a given issue may differ depending on the facts of each case and the position of the parties. Over time, findings on certain key issues have begun to crystallize into general principles that can serve as helpful guidance for organizations. In an effort to summarize the general principles that have emerged from court decisions and the Commissioner's findings to date, the OPC issues Interpretations of certain key concepts in PIPEDA. These Interpretations are not binding legal interpretations, but rather, are intended as a guide for compliance with PIPEDA. As the Commissioner issues more findings, and the courts render more decisions, these Interpretations may evolve and be further refined over time.

46. Policy Engagement Network. **"Electronic Health Privacy and Security in Developing Countries and Humanitarian Operations,"** London; 2011. Available at: https://www.privacyinternational.org/reports/medical-privacy-and-security-in-developing-countries-and-emergency-situations.

Expertise on the privacy and security aspects of the eHealth systems being deployed in resource-constrained environments such as developing countries and humanitarian operations is severely lacking; the knowledge base in this space is similarly weak. To be effective, the principles and aspirations for medical privacy enshrined in international agreements, policies and commitments must be supported by a local awareness of privacy responsibilities, a strong national legal and regulatory footing, and the appropriate use of information and communications technology. Among the legal and regulatory requirements for strong privacy and security protections are respect for self-determination, the appropriate and proportionate collection, management, access and disclosure of medical information, and strong mechanisms for monitoring compliance and accountability. Any solutions to medical privacy or health information security in these contexts will need to incorporate both technological means such as directed identifiers, access controls and encryption, as well as appropriate organizational, legal and policy responses. Any decision by funders, designers or implementers to exclude these privacy and security mechanisms from an eHealth system must be made as the result of informed deliberation rather than as a matter of expediency. This report was prepared by the Policy Engagement Network for the International Development Research Centre.

47. *RTI Rating Data Analysis Series :* **"Overview of Results and Trends,"** 2013. Available at: http://www.rti-rating.org/files/docs/Report.13.09.Overview of RTI Rating.pdf.

The RTI Rating, developed by Access Info Europe (AIE)2 and the Centre for Law and Democracy (CLD), 3 is a methodology which provides a numerical assessment or rating for the overall legal framework for the right to information (RTI) in a country, based on how well that framework gives effect to the right to access information held by public authorities. The methodology was first launched on International Right to Know Day, 28 September, in 2010, and comprehensive ratings of national RTI laws were provided in 2011 and then updated in 2012 and 2013.

48. Schwartz PM, Solove DJ. The PII Problem: **"Privacy and a New Concept of Personally Identifiable Information,"** *New York Univ Law Rev*. 2011;86:1814-1894. doi:10.2139/ssrn.1909366. http://ssrn.com/abstract=1909366

Personally identifiable information (PII) is one of the most central concepts in information privacy regulation. The scope of privacy laws typically turns on whether PII is involved. The basic assumption behind the applicable laws is that if PII is not involved, then there can be no privacy harm. At the same time, there is no uniform definition of PII in information privacy law. Moreover, computer science has shown that in many circumstances non-PII can be linked to individuals, and that de-identified data can be re-identified. PII and non-PII are thus not immutable categories, and there is a risk that information deemed non-PII at one time can be transformed into PII at a later juncture. Due to the malleable nature of what constitutes PII, some commentators have even suggested that PII be abandoned as the mechanism by which to define the boundaries of privacy law. In this Article, we argue that although the current approaches to PII are flawed, the concept of PII should not be abandoned. We develop a new approach called "PII 2.0," which accounts for PII's malleability. Based upon a standard rather than a rule, PII 2.0 utilizes a continuum of risk of identification. PII 2.0 regulates information that relates to either an "identified" or "identifiable" individual, and it establishes different requirements for each category. To illustrate this theory, we use the example of regulating behavioral marketing to adults and children. We show how existing approaches to PII impede the effective regulation of behavioral marketing, and how PII 2.0 would resolve these problems.

49. Singh Jack C, Mars Y, M. **"Pitfalls in computer housekeeping by doctors and nurses in KwaZulu-Natal: no malicious intent,"** *BMC Med Ethics*. 2013;14 Suppl 1(Suppl 1):S8. doi:10.1186/1472-6939-14-S1-S8. http://www.pubmedcentral.nih.gov/articlerender.fcgi?artid=3878337&tool=pmcentrez&rendertype=abstract

Information and communication technologies are becoming an integral part of medical practice, research and administration and their use will grow as telemedicine and electronic medical record use become part of routine practice. Security in maintaining patient data is important and there is a statuary obligation to do so, but few health professionals have been trained on how to achieve this. There is no information on the use of computers and email by doctors and nurses in South Africa in the workplace and at home, and whether their current computer practices meets legal and ethical requirements. The aims of this study were to determine the use of computers by healthcare practitioners in the workplace and home; the use and approach to data storage, encryption and security of patient data and patient email; and the use of informed consent to transmit data by email.

Data Release Policy

50. Boonyarattaphan A, Bai Y, Chung S. **"A security framework for e-Health service authentication and e-Health data transmission," In**: *2009 9th International Symposium on Communications and Information Technology, ISCIT 2009*.; 2009:1213-1218. doi:10.1109/ISCIT.2009.5341116. (To purchase this article please use the following link: http://ieeexplore.ieee.org/xpl/login.jsp?tp=&arnumber=5341116&url=http%3A%2F%2Fieeexplore.ieee.org%2Fxpls%2Fabs_all.jsp%3Farnumber%3D5341116)

e-Health services are gaining popularity due to largely reduced cost and wider range of advanced services. Securing e-health data transmission is challenging due to both (a) the criticality of personal medical information on effective medical treatment and privacy protection and (b) the significant computational needs of encryption algorithms. In this paper, a cost-effective security framework that suits for e-health authentication and data transmission is presented. The framework consists of efficient protocol architecture for e-health service; two risk adaptive authentication techniques and different encryption algorithms to handle data with different levels of importance, thus achieving required security requirements of e-health applications and reduction in the computational complexity and delay in e-health communication.

51. CDC and Agency for Toxic Substances and Disease Registry "**CDC/ATSDR Policy on Releasing and Sharing Data,"** 2003
http://www.cdc.gov/od/foia/policies/sharing.htm

The document contains policy on data release and sharing that balances the desire to disseminate data as broadly as possible with the need to maintain high standards for protection of sensitive information. The policy also ensures that CDC is in full compliance with the Health Insurance Portability and Accountability Act of 1996 (HIPAA),(where applicable) the Freedom of Information Act [FOIA], and the Office of Management and Budget Circular A110, and the Information Quality Guidelines.

52. CDC and Agency for Toxic Substances and Disease Registry "**CDC - CSTE Intergovernmental Data Release Guidelines Working Group (DRGWG),"** 2005, 86 p.
http://www.cdc.gov/od/foia/policies/drgwg.pdf or
http://www.cdc.gov/nchs/about/policy/policy.htm

This report contains16 guidelines and 6 procedures for implementing the "CDC/ATSDR Policy on Releasing and Sharing Data." Most guidelines (each represents a minimum standard for CDC programs) and procedures are accompanied by a best practice statement. Not HIV specific.

53. Canada – Alberta government *"Freedom of Information and Protection of Privacy (FOIP) Act,"* 2009, 66p. http://www.servicealberta.ca/foip/resources/guidelines-and-practices.cfm (the entire Act) http://www.servicealberta.ca/foip/resources/chapter-7.cfm (Rules and practices)

Provides a comprehensive source of reference on the application of the FOIP legislation by public bodies in Alberta. It interprets the Act and Regulation with reference to rulings by the Information and Privacy Commissioner. It also sets out roles and responsibilities and offers guidance on approaches and procedures that are intended to assist in the effective administration of the Act. Chapter 7 is dedicated to protection of privacy.

54. Curtis A, Mills JW, Agustin L, Cockburn M. **"Confidentiality risks in fine scale aggregations of health data,"** *Comput Environ Urban Syst*. 2011;35:57-64. doi:10.1016/j.compenvurbsys.2010.08.002. (To purchase this article please use the following link: http://www.sciencedirect.com/science/journal/01989715/35/1)

Spatial confidentiality concerns limit the sharing of data between health data guardians and other researchers. This reduces the contribution GIScience might play in understanding spatial patterns of poor health. This paper takes a first step towards easing data sharing by investigating the confidentiality risks in releasing aggregated data at a fine spatial resolution. A randomly generated cancer map is exported as a graduated color overlay to Google Earth and test subjects are asked to locate where they believe the disease cases reside. Risk is measured by both the separating distance and the number of alternate parcels between the " choice" and a randomly generated disease case. The paper also develops a simulation approach that can be used to test the level of risk involved with these choices. Results suggest that across the scales of aggregation tested in this paper, the finest of which is a 0.5. km grid, there was relatively little risk in revealing sensitive information. In addition, the closest student choice to a disease case was not consistent across aggregations, suggesting no underlying geographic vulnerability. Although the results presented here are encouraging, a series of subsequent investigations are needed before data sharing guidelines can be proposed.

55. Ganguly S, Kataria P, Juric R, Ertas A, Tanik MM. **"Sharing information and data across heterogeneous e-health systems,"** *Telemed J E Health*. 2009;15(5):454-64. doi:10.1089/tmj.2008.0149. (To purchase this article please use the following link:

http://www.ncbi.nlm.nih.gov/pubmed/19548826)

Information and data sharing across heterogeneous e-health systems, focusing on the management of patient care, have become the backbone of modern delivery of sustainable telemedicine services. Information and data available to healthcare practitioners in such environments range from patient's medical records, stored in repositories at places where patients have been treated, to a variety of information related to medical research, pharmaceutical products, or information stored within social networks of healthcare interest groups. This study sought to demonstrate two different approaches enabling the sharing of information/data across heterogeneous e-health systems: (1) Context-Aware Data Retrieval Architecture (CADRA), which secures the extraction and presentation of e-health information to users in requested format, and (2) Generic Ontology for Context Aware, Interoperable, and Data Sharing (Go-CID) software applications, which secure semantic interoperation across heterogeneous e-health data sources. Proof-of-concept was demonstrated in both cases, CADRA and Go-CID, to achieve understanding and building of knowledge about e-health environments. This study invites practical solutions for interoperable e-health systems.

56. Ihle P. **"Data protection and methodological aspects in compiling a routine database from statutory health insurance data for research purposes,"** *Bundesgesundheitsblatt Gesundheitsforschung Gesundheitsschutz*. 2008;51:1127-1134. doi:10.1007/s00103-008-0647-x. (To purchase this article please use the following link: http://www.ncbi.nlm.nih.gov/pubmed/18985406)

Personally identifiable routine data generated by the SHI (statutory health insurance) offer inexpensive and large amounts of data gathered over long periods of observation for use in numerous fields of application including health services research and epidemiology of health care. As a source of medical health information, these data are subject to particular EU data protection directives according to which they can only be used under certain conditions and following careful consideration of the various interests involved. These interests include the protection of personal privacy, on the one hand, and the freedom of research, on the other. As personally identifiable data, these data are fully subject to general and specific data privacy regulations, such as the consideration of intended use; the specification of forms of data processing, duration of use, and group of users; and the development of a data protection concept. If primary data are additionally collected, the patient is to be fully informed about the intended contents of analysis and the use of his/her data in order that informed consent can be provided. Methodological standards such as the verification of completeness and plausibility are also to be met when compiling an insuree database.

57. Juengst ET. **"TMI! Ethical challenges in managing and using large patient data sets,"** *N C Med J*. 1996;75(3):214-7. (To purchase this article please use the following link: http://www.ncbi.nlm.nih.gov/pubmed/24830499)

The advent and expansion of electronic medical record systems and open-access databases are creating a "data tsunami." As this wave descends, we must anticipate and address several ethical and social risks: threats to patient privacy, threats to the reputations of various social groups, and threats to public trust in biomedical research.

58. Matthews GJ, Harel O. **"Data confidentiality: A review of methods for statistical disclosure limitation and methods for assessing privacy,"** *Stat Surv*. 2011;5:1-29. doi:10.1214/11-SS074. http://projecteuclid.org/euclid.ssu/1296828958

There is an ever-increasing demand from researchers for access to useful microdata files. However, there are also growing concerns regarding the privacy of the individuals contained in the microdata. Ideally, microdata could be released in such a way that a balance between usefulness of the data and privacy is struck. This paper presents a review of proposed methods of statistical disclosure control and techniques for assessing the privacy of such methods under different definitions of disclosure.

59. Narayanan A, Shmatikov V. **“Myths and fallacies of “personally identifiable information.”** *Commun ACM*. 2010;53(6):24. doi:10.1145/1743546.1743558. http://portal.acm.org/citation.cfm?doid=1743546.1743558

This article talks about the technical and legal meanings of “personally identifiable information” (PII) and argues that the term means next to nothing and must be greatly de-emphasized, if not abandoned, in order to have a meaningful discourse on data privacy. Reidentification of Massachusetts hospital records showed that naive deidentification via PII removal can be reversed. That led to a cat-and-mouse game between deidentification and reidentification, with standards such as HIPAA mandating removal of a more comprehensive set of attributes. In parallel, techniques for data transformations that enable specific categories of computations in a mathematically rigorous privacy-preserving way were developed — “differential privacy” enables sidestepping the need for anonymization altogether. Given the increasingly easy availability of public “auxiliary information” about individuals (e.g., from social media), is it possible to provide any technical privacy guarantees via anonymization, while maintaining data utility? How identifiable are people’s footprints in the rich “longitudinal” databases that are common today? Can we characterize which types of data can lead to reidentification, thus salvaging the notion of “Personally Identifiable Information?” Finally, how many bits of uncorrelated information (“entropy”) are required to reidentify individuals in large datasets?

60. Narayanan A, Shmatikov V. **“De-anonymizing social networks,”** *Secur Privacy, 2009 30th* IEEE Symposium on 17-20 May 2009. doi:10.1109/SP.2009.22. (To purchase this article please use the following link: http://ieeexplore.ieee.org/xpls/abs_all.jsp?arnumber=5207644)

Operators of online social networks are increasingly sharing potentially sensitive information about users and their relationships with advertisers, application developers, and data-mining researchers. Privacy is typically protected by anonymization, i.e., removing names, addresses, etc. We present a framework for analyzing privacy and anonymity in social networks and develop a new re-identification algorithm targeting anonymized social- network graphs. To demonstrate its effectiveness on real- world networks, we show that a third of the users who can be verified to have accounts on both Twitter, a popular microblogging service, and Flickr, an online photo-sharing site, can be re-identified in the anonymous Twitter graph with only a 12% error rate. Our de-anonymization algorithm is based purely on the network topology, does not require creation of a large number of dummy “sybil” nodes, is robust to noise and all existing defenses, and works even when the overlap between the target network and the adversary’s auxiliary information is small.

61. Neame R. **“Effective sharing of health records, maintaining privacy: a practical schema,”** *Online J Public Health Inform*. 2013;5(2):217. doi:10.5210/ojphi.v5i2.4344. http://www.pubmedcentral.nih.gov/articlerender.fcgi?artid=3733761&tool=pmcentrez&rendertype=abstract

A principal goal of computerisation of medical records is to join up care services for patients, so that their records can follow them wherever they go and thereby reduce delays, duplications, risks and errors, and costs. Healthcare records are increasingly being stored electronically, which has created the necessary conditions for them to be readily sharable. However simply driving the implementation of electronic medical records is not sufficient, as recent developments have demonstrated (1): there remain significant obstacles. The three main obstacles relate to (a) record accessibility (knowing where event records are and being able to access them), (b) maintaining privacy (ensuring that only those authorised by the patient can access and extract meaning from the records) and (c) assuring the functionality of the shared information (ensuring that the records can be shared non-proprietorially across platforms without loss of meaning, and that their authenticity and trustworthiness are demonstrable). These constitute a set of issues that need new thinking, since existing systems are struggling to deliver them. The solution to this puzzle lies in three main parts. Clearly there is only one

environment suited to such widespread sharing, which is the World Wide Web, so this is the communications basis. Part one requires that a sharable synoptic record is created for each care event and stored in standard web-format and in readily accessible locations, on 'the web' or in 'the cloud'. To maintain privacy these publicly-accessible records must be suitably protected either stripped of identifiers (names, addresses, dates, places etc.) and/or encrypted: either way the record must be tagged with a tag that means nothing to anyone, but serves to identify and authenticate a specific record when retrieved. For ease of retrieval patients must hold an index of care events, records and web locations (plus any associated information for each such as encryption keys, context etc.). For added security, as well as for trustworthiness, a method of verifying authenticity, integrity and authorship is required, which can be provided using a public key infrastructure (PKI) for cryptography (2). The second part of the solution is to give control over record access and sharing to the patient (or their identified representative), enabling them to authorise access by providing the index and access keys to their records. This can be done using a token (fe.g. smart card) or a secure online index which holds these details: this serves to relieve the formal record keeper of responsibility for external access control and privacy (internal access control and privacy can remain an institutional responsibility). The third part of the solution is to process the content of the stored records such that there is a 'plain English' copy, as well as an electronic copy which is coded and marked up using XML tags for each data element to signify 'type' (e.g. administrative, financial, operational, clinical etc.) and sub-types (e.g. diagnosis, medication, procedure, investigation result etc.). This ensures that the recipient can always read the data using a basic browser, but can readily manipulate and re-arrange the data for display and storage if they have a more sophisticated installation.

62. Ribeiro LS, Viana-Ferreira C, Oliveira JL CC. **"XDS-I Outsourcing Proxy: Ensuring Confidentiality While Preserving Interoperability,"** *PubMed*. 2014; IEEE J Biomed Health Inform. 2014 Jul;18(4):1404-12. doi: 10.1109/JBHI.2013.2292776. (To purchase this article please use the following link: http://www.ncbi.nlm.nih.gov/pubmed/25014941)

The interoperability of services and the sharing of health data have been a continuous goal for health professionals, patients, institutions, and policy makers. However, several issues have been hindering this goal, such as incompatible implementations of standards (e.g., HL7, DICOM), multiple ontologies, and security constraints. Cross-enterprise document sharing (XDS) workflows were proposed by Integrating the Healthcare Enterprise (IHE) to address current limitations in exchanging clinical data among organizations. To ensure data protection, XDS actors must be placed in trustworthy domains, which are normally inside such institutions. However, due to rapidly growing IT requirements, the outsourcing of resources in the Cloud is becoming very appealing. This paper presents a software proxy that enables the outsourcing of XDS architectural parts while preserving the interoperability, confidentiality, and searchability of clinical information. A key component in our architecture is a new searchable encryption (SE) scheme-Posterior Playfair Searchable Encryption (PPSE)-which, besides keeping the same confidentiality levels of the stored data, hides the search patterns to the adversary, bringing improvements when compared to the remaining practical state-of-the-art SE schemes.

Electronic Security

63. Akinyele J a, Lehmann CU, Green MD, Pagano MW, Peterson ZNJ, Rubin AD. **"Self-Protecting Electronic Medical Records Using Attribute-Based Encryption,"** *ePrint IACR org*. 2010;1:1-20. Available at: http://eprint.iacr.org/2010/565.

We provide a design and implementation of self-protecting electronic medical records (EMRs) using attribute-based encryption. Our system allows healthcare organizations to export EMRs to storage locations outside of their trust boundary, including mobile devices, Regional Health Information Organizations (RHIOs), and cloud systems such as Google Health. In contrast to some previous approaches to this problem, our solution is designed to maintain EMR availability even when providers are offline, i.e., where network connectivity is not available (for example, during a natural disaster). To balance the needs of emergency care and patient privacy, our system is designed to provide for fine-grained encryption and is able to protect individual items within an EMR, where each encrypted item may have its own access control

policy. To validate our architecture, we implemented a prototype system using a new dual-policy attribute-based encryption library that we developed. Our implementation, which includes an iPhone app for storing and managing EMRs offline, allows for flexible and automatic policy generation. An evaluation of our design shows that our ABE library performs well, has acceptable storage requirements, and is practical and usable on modern smartphones.

64. Bajaj S, Sion R. TrustedDB: **"A Trusted Hardware based Database with Privacy and Data Confidentiality,"** In: *Proceedings of the 2011 International Conference on Management of Data - SIGMOD '11*.; 2011:205-216. doi:10.1145/1989323.1989346. (To purchase this article please use the following link: http://portal.acm.org/citation.cfm?doid=1989323.1989346)

TrustedDB is an outsourced database prototype that allows clients to execute SQL queries with privacy and under regulatory compliance constraints without having to trust the service provider. TrustedDB achieves this by leveraging server-hosted tamper-proof trusted hardware in critical query processing stages. TrustedDB does not limit the query expressiveness of supported queries. And, despite the cost overhead and performance limitations of trusted hardware, the costs per query are orders of magnitude lower than any (existing or) potential future software-only mechanisms. TrustedDB is built and runs on actual hardware, and its performance and costs are evaluated here.

65. Cheswick W. R., Bellovin S. M., and Rubin A. D. **"Firewalls and Internet Security: Repelling the Wily Hacker,"** 2003, 464 p. Addison-Wesley Professional Computing Series http://www.wilyhacker.com

This technical book is written primarily for the network administrator who must protect an organization from unhindered exposure to the Internet. It contains the following sections: Security review, Threats, Safer tools and Services, Firewalls and VPNs, Protecting an organization, Lessons Learned, Appendix on Cryptography.

66. Du W. SEED: **"Hands-on lab exercises for computer security education,"** *IEEE Secur Priv*. 2011;9:70-73. doi:10.1109/MSP.2011.139. http://www.cis.syr.edu/~wedu/Research/paper/seed_ieeeSPmagazine2011.pdf

The 30 SEED (Security Education) labs cover such topics as vulnerabilities, attacks, software security, system security, network security, Web security, access control, authentication, and cryptography. More than 80 universities have requested the instructor's manual and have adopted a selection of SEED labs for their courses.

67. Frellick M. **"New biometric device aims to improve record-keeping and security,"** *PubMed*. 2013. (To purchase this article please use the following link: http://www.ncbi.nlm.nih.gov/pubmed/23617111)

The article reports on biometric devices called palm readers which are used in many U.S. hospitals to improve record keeping and security and capture near-infrared images of the veins in a patient's hand and automatically link to the correct electronic patient record. A discussion of several health care facilities, including Harris Health System in Texas and New York University's Langone Medical Center, that have implemented the devices, is presented.

68. Hallam K. **"Biometric Technology Combats Medical Identity Theft,"** *BusinessWeek.com*. 2013:1. Available at: http://search.ebscohost.com/login.aspx?direct=true&db=buh&AN=87676111&site=ehost-live.

The article reports on the fight of U.S. health care industry against medical identity theft by using biometric technology to verify patient identities. It mentions that the fraud will cost health care providers billions annually and the demand is growing for biometric devices such as iris scanners and palm vein readers. It notes that the iris is considered the best biometric identifier

and is 100,000 times more resistant to false identification than facial-recognition software.

69. International Organization for Standardization **"ISO/IEC 27001:2013 Information technology - Security techniques - Code of practice for information security controls,"** 2013 http://www.iso27001security.com/html/27001.html (access requires payment)

ISO/IEC 27001 formally specifies an Information Security Management System (ISMS), a suite of activities concerning the management of information security risks. The ISMS is an overarching management framework through which the organization identifies, analyzes and addresses its information security risks. The ISMS ensures that the security arrangements are fine-tuned to keep pace with changes to the security threats, vulnerabilities and business impacts. The standard covers all types of organizations (e.g. commercial enterprises, government agencies, non-profits), all sizes (from micro-businesses to huge multinationals), and all industries or markets (e.g. retail, banking, defense, healthcare, education and government). This is clearly a very wide brief than ISO/IEC 17799:2005.

70. Jan H. **"Anonymous authentication for smartcards,"** *Radioengineering*. 2010;19(2):363-368. Available at: http://radioeng.cz/fulltexts/2010/10_02_363_368.pdf

The paper presents an innovative solution in the field of RFID (Radio-Frequency IDentification) smartcard authentication. Currently the smartcards are used for many purposes - e.g. employee identification, library cards, student cards or even identity credentials. Personal identity is revealed to untrustworthy entities every time we use these cards. Such information could later be used without our knowledge and for harmful reasons like shopping pattern scanning or even movement tracking. We present a communication scheme for keeping one's identity private in this paper. Although our system provides anonymity, it does not allow users to abuse this feature. The system is based on strong cryptographic primitives that provide features never available before. Besides theoretical design of the anonymous authentication scheme and its analysis we also provide implementation results.

71. Krishnamurthy B, Wills CE. **"On the leakage of personally identifiable information via online social networks,"** *ACM SIGCOMM Comput Commun Rev*. 2010;40(1):112. (To purchase this article please use the following link: http://dl.acm.org/citation.cfm?id=1397744)

For purposes of this paper, we define "Personally identifiable information" (PII) as information which can be used to distinguish or trace an individual's identity either alone or when combined with other information that is linkable to a specific individual. The popularity of Online Social Networks (OSN) has accelerated the appearance of vast amounts of personal information on the Internet. Our research shows that it is possible for third-parties to link PII, which is leaked via OSNs, with user actions both within OSN sites and else- where on non-OSN sites. We refer to this ability to link PII and combine it with other information as "leakage". We have identified multiple ways by which such leakage occurs and discuss measures to prevent it.

72. Li M, Yu S, Zheng Y, Ren K, Lou W. **"Scalable and secure sharing of personal health records in cloud computing using attribute-based encryption,"** *IEEE Trans Parallel Distrib Syst*. 2013;24:131-143. doi:10.1109/TPDS.2012.97. http://www.acsu.buffalo.edu/~kuiren/Li_TPDS_2012.pdf

Personal health record (PHR) is an emerging patient-centric model of health information exchange, which is often outsourced to be stored at a third party, such as cloud providers. However, there have been wide privacy concerns as personal health information could be exposed to those third party servers and to unauthorized parties. To assure the patients' control over access to their own PHRs, it is a promising method to encrypt the PHRs before outsourcing. Yet, issues such as risks of privacy exposure, scalability in key management, flexible access, and efficient user revocation, have remained the most important challenges

toward achieving fine-grained, cryptographically enforced data access control. In this paper, we propose a novel patient-centric framework and a suite of mechanisms for data access control to PHRs stored in semitrusted servers. To achieve fine-grained and scalable data access control for PHRs, we leverage attribute-based encryption (ABE) techniques to encrypt each patient's PHR file. Different from previous works in secure data outsourcing, we focus on the multiple data owner scenario, and divide the users in the PHR system into multiple security domains that greatly reduces the key management complexity for owners and users. A high degree of patient privacy is guaranteed simultaneously by exploiting multiauthority ABE. Our scheme also enables dynamic modification of access policies or file attributes, supports efficient on-demand user/attribute revocation and break-glass access under emergency scenarios. Extensive analytical and experimental results are presented which show the security, scalability, and efficiency of our proposed scheme.

73. National Institute of Standards and Technology "**Guideline on network security testing,"** 2003, 92 p. http://csrc.nist.gov/publications/nistpubs/800-42/NIST-SP800-42.pdf

This detailed overview contains names of many tools, for example, a list of firewall product names. Not HIV or health specific.

74. National Institute of Standards and Technology "**An introductory Resource Guide for Implementing the HIPAA Security rule,"** 2005, 137 p. http://csrc.nist.gov/publications/nistpubs/800-66/SP800-66.pdf

The guide identifies resources relevant to the specific security standards included in the HIPAA security rule and provides implementation examples for each. It focuses on the safeguarding of electronic protected health information.

75. National Institute of Standards and Technology "**Security Self-Assessment Guide for Information Technology Systems, revised2005,"** 2005 http://csrc.nist.gov/publications/nistpubs/800-26/Mapping-of-800-53v1.doc

This technical document provides a comprehensive security self assessment questionnaire. It includes more than 200 controls: management controls, operational controls, and technical controls. Not HIV or health specific.

76. Ohno-Machado L. **"Sharing data for the public good and protecting individual privacy: informatics solutions to combine different goals,"** *J Am Med Inform Assoc*. 2013;20:1. doi:10.1136/amiajnl-2012-001513. http://www.pubmedcentral.nih.gov/articlerender.fcgi?artid=3555342&tool=pmcentrez&rendertype=abstract

The article introduces several reports within the issue about privacy technology in medicine, including an article about patient phenotyping data by Hripcsak, an article about biomedical knowledge computing by Farley, and an article by Kawamoto about knowledge sharing in clinical decision support.

77. Plaga R. Biometric keys: **"Suitable use cases and achievable information content,"** *Int J Inf Secur*. 2009;8:447-454. doi:10.1007/s10207-009-0090-5. (To purchase this article please use the following link: http://connection.ebscohost.com/c/articles/44793175/biometric-keys-suitable-use-cases-achievable-information-content)

This article surveys use cases for cryptographic keys extracted from biometric templates ("biometric keys"). It lays out security considerations that favor uses for the protection of the confidentiality and privacy of biometric information itself. It is further argued that the cryptographic strength of a biometric key is determined by its true information content. I

propose an idealized model of a biometric system as a Shannon channel. The information content that can be extracted from biometric templates in the presence of noise is determined within this model. The performance of state-of-the-art biometric technology to extract a key from a single biometric feature (like, e.g., one iris pattern or one fingerprint) is analyzed. Under reasonable operating conditions the channel capacity limits the maximal achievable information content k of biometric key to values smaller than about 30 bits. This upper length limit is too short to thwart "brute force" attacks on crypto systems employing biometric keys. The extraction of sufficiently long biometric keys requires either: (a) technological improvements that improve the recognition power of biometric systems considerably or (b) the employment of multimodal and/or multiinstance biometrics or (c) the use of novel biometric features, such as, e.g., the pattern DNA nucleotides in the human genome.

78. Puttaswamy KPN, Kruegel C, Zhao BY. *Silverline :* **"Toward Data Confidentiality in Third-Party Clouds,"** 2010. (To purchase this article please use the following link: http://128.111.41.37/research/tech_reports/abstract.php?id=1000)

By offering high availability and elastic access to resources, third party cloud infrastructures such as Amazon AWS and Microsoft Azure are revolutionizing the way todays businesses operate. Unfortunately, taking advantage of their benefits requires businesses to accept a number of serious risks to data security. Factors such as software bugs, operator errors and external attacks can all compromise the confidentiality of sensitive data on external clouds, making them vulnerable to unauthorized access by malicious parties. In this paper, we study and seek to improve the confidentiality of application data stored on third-party computing clouds. We propose to identify and encrypt all functionally encryptable data, sensitive data that can be encrypted without limiting the functionality of the cloud service. Such data would only be stored on the cloud in an encrypted form, accessible only to users with the correct keys, thus ensuring its confidentiality against unintentional errors and attacks alike. We describe Silverline, a set of tools that automatically 1) identify all functionally encryptable data in a cloud application, 2) assign encryption keys to specific data subsets to minimize key management complexity while ensuring robustness to key compromise, and 3) provide transparent data access at the user device while preventing key compromise even from malicious clouds. Through experiments with real applications, we find that many web applications are dominated by data sharing components that do not require access to raw data. Thus, Silverline can protect the vast majority of data on these applications, simplify key management, and protect against key compromise. Together, our techniques provide a substantial first step towards simplifying the complex process of incorporating data confidentiality into cloud applications.

79. Sokolova M, El Emam K, Arbuckle L, Neri E, Rose S, Jonker E. **"P2P Watch: Personal Health Information Detection in Peer-to-Peer File-Sharing Networks,"** *J Med Internet Res*. 2012;14:e95. doi:10.2196/jmir.1898. (To purchase this article please use the following link: http://www.ncbi.nlm.nih.gov/pubmed/22776692)

Users of peer-to-peer (P2P) file-sharing networks risk the inadvertent disclosure of personal health information (PHI). In addition to potentially causing harm to the affected individuals, this can heighten the risk of data breaches for health information custodians. Automated PHI detection tools that crawl the P2P networks can identify PHI and alert custodians. While there has been previous work on the detection of personal information in electronic health records, there has been a dearth of research on the automated detection of PHI in heterogeneous user files.

80. Scarfone K, Hoffman P. **"Guidelines on firewalls and firewall policy: recommendations of the National Institute of Standards and Technology,"** NIST Spec Publ. 2009:74. http://scholar.google.com/scholar?hl=en&btnG=Search&q=intitle:Guidelines+on+Firewalls+and+Firewall+Policy+Recommendations+of+the+National+Institute+of+Standards+and+Technology#1

This document provides introductory information about firewalls and firewall policy primarily to assist those responsible for network security. It addresses concepts relating to the design,

selection, deployment, and management of firewalls and firewalls environments. Not HIV or health specific.

2.

81. Schneier B. "**Secrets and Lies: Digital Security in a Networked World,"** 2004 John Wiley and Sons, ISBN**:** 0471453803

Describes, using concise everyday analogies, the strategies hackers use to compromise electronic data systems.

82. Yau S, An H. **"Confidentiality Protection in Cloud Computing Systems,"** *Int J Softw Informatics*. 2010;4(4):351-365. Available at: http://scholar.google.com/scholar?hl=en&btnG=Search&q=intitle:Confidentiality+Protection+in+Cloud+Computing+Systems#0

Current cloud computing systems pose serious limitation to protecting users' data confidentiality. Since users' sensitive data is presented in unencrypted forms to remote machines owned and operated by third party service providers, the risks of unauthorized disclosure of the users' sensitive data by service providers may be high. Many techniques for protecting users' data from outside attackers are available, but currently there exists no effective way for protecting users' sensitive data from service providers in cloud computing. In this paper, an approach is presented to protecting the confidentiality of users' data from service providers, and ensures that service providers cannot access or disclose users' confidential data being processed and stored in cloud computing systems. Our approach has three major aspects: 1) separating software service providers and infrastructure service providers in cloud computing, 2) hiding information of the owners of data, and 3) data obfuscation. An example to show how our approach can protect the confidentiality of users' data from service providers in cloud computing is given. Experimental results are presented to show that our approach has reasonable performance.

83. Zhou X, Peng B, Li YF, Chen Y, Tang H, Wang X. **"To release or not to release: Evaluating information leaks in aggregate human-genome data,"** In: *Lecture Notes in Computer Science (including Subseries Lecture Notes in Artificial Intelligence and Lecture Notes in Bioinformatics)*.Vol 6879 LNCS.; 2011:607-627. doi:10.1007/978-3-642-23822-2_33. http://www.cs.indiana.edu/~zhou/files/release.pdf

The rapid progress of human genome studies leads to a strong demand of aggregate human DNA data (e.g, allele frequencies, test statistics, etc.), whose public dissemination, however, has been impeded by privacy concerns. Prior research shows that it is possible to identify the presence of some participants in a study from such data, and in some cases, even fully recover their DNA sequences. A critical issue, therefore, becomes how to evaluate such a risk on individual data-sets and determine when they are safe to release. In this paper, we report our research that makes the first attempt to address this issue. We first identified the space of the aggregate-data-release problem, through examining common types of aggregate data and the typical threats they are facing. Then, we performed an in-depth study on different scenarios of attacks on different types of data, which sheds light on several fundamental questions in this problem domain. Particularly, we found that attacks on aggregate data are difficult in general, as the adversary often does not have enough information and needs to solve NP-complete or NP-hard problems. On the other hand, we acknowledge that the attacks can succeed under some circumstances, particularly, when the solution space of the problem is small. Based upon such an understanding, we propose a risk-scale system and a methodology to determine when to release an aggregate data-set and when not to. We also used real human-genome data to verify our findings. © 2011 Springer-Verlag.

Additional Material

Overall

84. Anderson RJ, University of Cambridge "**Security in clinical information systems,**" 1996 http://www.cl.cam.ac.uk/users/rja14/policy11/policy11.html

Describes threats to confidentiality, integrity, and availability of personal health information in the light of experience in the UK and overseas, and proposes a clinical information security policy that enables the principle of patient consent to be enforced in the kind of heterogeneous distributed system currently under construction in the UK. An information security policy says who may access what information; access includes such activities as reading, writing, appending, and deleting data.

85. Dr Peter Drury, eHealth International "**eHealth: a model for developing countries,**" 2005, p8 http://www.ehealthinternational.net/

This paper proposes a model or framework for analysis, to inform the development of eHealth in developing countries. The framework has five components – the 5Cs: context of poverty, content of health information provided to health workers, connectivity within and between health facilities, building workforce capacity, supporting community development.

86. Kaiser K. **"Protecting Respondent Confidentiality in Qualitative Research,"** *Qual Health Res*. 2009;19(11):1632-1641. doi:10.1177/1049732309350879 http://www.ncbi.nlm.nih.gov/pmc/articles/PMC2805454/pdf/nihms162528.pdf

For qualitative researchers, maintaining respondent confidentiality while presenting rich, detailed accounts of social life presents unique challenges. These challenges are not adequately addressed in the literature on research ethics and research methods. Using an example from a study of breast cancer survivors, I argue that by carefully considering the audience for one's research and by re-envisioning the informed consent process, qualitative researchers can avoid confidentiality dilemmas that might otherwise lead them not to report rich, detailed data.

87. Office of the Saskatchewan Information and Privacy Commissioner **"Glossary of Common Terms The Health Information Protection Act (HIPA),"** 2009. http://www.oipc.sk.ca/Resources/HIPA Glossary - Blue Box.pdf

What follows is a list of common terms used in the HIPA regime in Saskatchewan. A few of these terms are defined in section 2 of HIPA. Many of these terms have meanings that are well established in Canadian jurisprudence and in decisions/Orders of privacy oversight agencies developed over the last 27 years.

88. Sarah B. Mcfarlane "**Harmonizing HIS with information systems in other social and economic sectors,**" 2005, 7 p. http://www.scielosp.org/pdf/bwho/v83n8/v83n8a12.pdf

A WHO bulletin about the needs of a better cross-coordination among social and economic sectors in order to optimize the HIS in low- and middle-income countries.

89. State of Texas Department of Information Resources "**Privacy Issues Involved in Electronic Government,**" 2000 http://www.dir.state.tx.us/taskforce/report/privacy.doc

Privacy and information held in e-governments: Overview, comparison among other countries, and recommendations for Texas.

90. The Academy of Medical Sciences **"Personal Data for Public Good: Using Health Information in Medical Research,"** 2006. http://www.acmedsci.ac.uk/policy/policy-projects/personal-data/

The document outlines AMS concerns, and makes recommendations for what they see as possible improvements to research practices. Reform is needed, according to the AMS, because data protection and other related legislation has often been interpreted by regulatory bodies in contradictory and confusing ways. Because of the resulting uncertainty, researchers are often advised that personal medical data cannot be used in their research studies unless there is consent from the individual or the data has been anonymised.

91. Zelazny F. **"The Evolution of India ' s UID Program Lessons Learned and Implications for Other Developing Countries CGD Policy Paper 008,"** 2012; (August). Available at: http://www.cgdev.org/sites/default/files/1426371_file_Zelazny_India_Case_Study_FINAL.pdf.

India has embarked on an ambitious new programme to provide its citizens and residents a unique, official identity. The universal identity programme aims to improve the delivery of government services, reduce fraud and corruption, facilitate robust voting processes, and improve security. It is by far the largest application of biometric identification technology to date and will have far-reaching implications for other developing countries that are looking to adopt national identity programmes to further social and economic development. This paper discusses the evolution of the universal identity programme, the innovative organization and path-breaking technology behind it, how it is being rolled out, and how robust identity is beginning to be used. The paper also draws lessons for other countries. Unlike many legacy national identity programmes, the universal identity programme is designed from the ground up to support authentication. Its use of multimodal biometrics increases inclusion into the main enrolment database and has a huge impact in improving accuracy. It relies on mobile technology but has also become a driving force behind the development of that technology. Its standards-based approach opens the way for vendor competition and cost reduction. At the same time, its exclusive focus on authentication still leaves the problem of how to validate certain aspects of identity, such as citizenship status. The paper discusses this in the context of the turf war between the universal.

Legal, ethical

92. Australian government "**"Federal Privacy Act, 1988, 2000,"**
http://www.privacy.gov.au/act/privacyact/index.html
"**Guidelines on Privacy in the Private Health Sector, 2001,"**
http://www.privacy.gov.au/health/guidelines/index.html#1
"**Information Technology and Internet Issues,"**
http://www.privacy.gov.au/internet/index.html
"**Guidelines Under Section 95 of the Privacy Act 1988, 2000,"**
http://www.privacy.gov.au/publications/e26.pdf

Highly readable but somewhat briefer coverage of the topics covered under "Recommended Readings, above.

93. Doyle, J. Lane, J. Theeuwes, L. Zayatz "**Confidentiality, Disclosure and Data Access: Theory and Practical Applications for Statistical Agencies,"** 2002 ELSEVIER 2, 462 pages, ISBN: 0-444-50761

Provides a review of new research in the area of confidentiality and statistical disclosure techniques. A major section of the book provides an overview of new advances in the field of both economic and demographic data in measuring disclosure risk and information loss. It also presents new information on the different approaches taken by statistical agencies in

disseminating data -- ranging from licensing agreements to secure access – and provides a new survey of which statistical disclosure techniques are used by statistical agencies around the world. This is complimented by a series of chapters on public perceptions of statistical agency actions, including the results of a new survey on business perceptions. The book

94. **Fairchild, Amy L. and Bayer, Ronald "Ethics and the Conduct of Public Health Surveillance,"** 2004 *Science* 30 January 2004:Vol. 303. no. 5658, pp. 631 – 632 http://www.sciencemag.org/cgi/content/summary/303/5658/631 *(accessible only by members)*

Efforts to distinguish between public health surveillance and epidemiological research are burdened by history. The authors of this policy forum say the moment is right to reframe the policy discussion and to recognize the imperative of ethical review of surveillance as well as research.

95. Joshua B. Bolten "**OMB Guidance for Implementing the Privacy Provisions of the E-Government Act of 2002,"** 2003 http://www.whitehouse.gov/omb/memoranda/m03-22.html

Provides information to agencies on implementing the privacy provisions of the E-Government Act of 2002

96. **Kathleen M. MacQueen and James W. Buehler "Ethics, Practice, and Research in Public Health,"** 2004 American Journal of Public Health June 2004, Vol 94, No. 6 | 928-931

http://www.ajph.org/cgi/content/abstract/94/6/928 *(accessible only by members)*
Ethical issues that can arise in distinguishing public health research from practice are highlighted in 2 case studies: 1) an investigation of a tuberculosis outbreak in a prison and 2) an evaluation of a program for improving HIV prevention services.

97. Protection and Advocacy System of New Mexico "**HIV and Your Legal Rights,"** 1996 http://www.aegis.com/law/journals/1996/LEGLBOOK.html

This booklet covers the inclusion of HIV/AIDS as a disability for the purposes of disability discrimination law; confidentiality; HIV testing and consent; insurance benefits; discrimination in housing, employment, medical care, and public accommodations; end-of-life planning; and disability and other public benefits. A frequently asked questions part includes answers to questions such as: "Can I be Denied Insurance Because I Have HIV?" or "Can I be Fired Or Not Hired Because I Have HIV/AIDS?"

98. U.K. Government "**Data Protection Act 1998,"** ISBN 0 10 542998 8 http://www.opsi.gov.uk/ACTS/acts1998/19980029.htm

*This Act gives individuals the right to access information held about them by organizations. The act governs how organizations can use the personal information that they hold - including how they acquire, store, share or dispose of it. The act is administered and enforced by the Information Commissioner - an independent authority who is appointed by the Queen and reports directly to parliament. Data protection is an international issue which results from European legislation (**Directive 95/46/EC, see R10)***

99. UNAIDS/WHO **"Guiding Principles on Ethical Issues in HIV Surveillance,"** UNAIDS / WHO Working Group 2013

http://apps.who.int/iris/bitstream/10665/90448/1/9789241505598_eng.pdf. *This publication provides guidance on the ethical issues that emerge in the context of surveillance. It was commissioned by the WHO/UNAIDS Surveillance Working Group to complement the discussions that take place with country staff in the course of training on second generation surveillance. The potential audience is therefore the epidemiologists and programme managers who are responsible for surveillance activities. The issues discussed here are, however, of interest to all health professionals concerned with the ethics of research on HIV.*

100. U.S. Government "**The E-Government Act of 2002,**"
http://www.whitehouse.gov/omb/egov/g-4-act.html
The Act, which has been made for governmental internet web-sites, contains these 2 chapters:
TITLE III: Information Security
TITLE V: Confidential Information Protection and Statistical Efficiency.

101. U.S. Public Law 104-191 "**Health Insurance Portability and Accountability Act of 1996,**"
http://www.cms.hhs.gov/HIPAAGenInfo/

The Administrative Simplification provisions of the Health Insurance Portability and Accountability Act of 1996 (HIPAA, Title II) require the Department of Health and Human Services (HHS) to establish national standards for electronic health care transactions and national identifiers for providers, health plans, and employers. It also addresses the security and privacy of health data. Adopting these standards will improve the efficiency and effectiveness of the nation's health care system by encouraging the widespread use of electronic data interchange in health care.

102. USAID "**USAID Compliance With the E-Government Act of 2002,**"
http://www.usaid.gov/policy/egov/

Outlines how USAID's public website complies with Federal information resource management law and policy as detailed in OMB Memorandum M-05-04.

Data Release Policies

103. Armstrong, Marc P.; Rushton, Gerard; Zimmerman, Dale L. "**Geographically Masking Health Data to Preserve Confidentiality,**" 1999 *Statistics in Medicine* 18, 497-525
http://www.uiowa.edu/~geog/faculty/armstrong/Masking.pdf

This document describes methods of geographically masking individual-level data.

104. Federal Committee on Statistical Methodology "**Confidentiality and Data Access Committee (CDAC),**" 1999 http://www.fcsm.gov/committees/cdac/index.html

CDAC is sponsored by the Federal Committee on Statistical Methodology to provide a forum for staff members of federal statistical agencies who work on confidentiality and data access topics to communicate among themselves, exchange ideas, etc. This site also contains the brochure, "Confidentiality and Data Access Issues Among Federal Agencies."

105. Federal Geographic Data Committee "**FGDC Policy on Access to Public Information and the Protection of Personal Information Privacy in Federal Geospatial Databases,**" 1998
http://www.fgdc.gov/library/factsheets/factsheets-biblio/privacy-factsheet

This policy articulates the FGDC's endorsement of public access to information and appropriate protections for the privacy and confidentiality of personal information in federal geospatial databases. The Federal Geographic Data *Committee (FGDC) is an interagency committee that promotes the coordinated development, use, sharing, and dissemination of geospatial* data *on a national basis. The Office of Management and Budget (OMB) established the FGDC in 1990.*

106. Goss, Jon, Department of Geography University of Hawai'i " '**We Know Who You Are and We Know Where You Live': The Instrumental Rationality of Geodemographic Systems,"** 1995 *Economic Geography* Vol. 71, No. 2., pp. 171-198

This paper provides a critique of geodemographic systems, sophisticated marketing tools that combine massive electronic data bases on consumer characteristics and behavior, segmentation schemes, and Geographic Information Systems (GIS).

107. Leah K. VanWey, Ronald R. Rindfuss, Myron P. Gutmann, Barbara Entwisle, and Deborah L. Balk. "**Spatial Demography Special Feature: Confidentiality and spatially explicit data: Concerns and challenges,"** PNAS 2005 102: 15337-15342 http://www.pnas.org/cgi/reprint/102/43/153377

Recent theoretical, methodological, and technological advances in the spatial sciences create an opportunity for social scientists to address questions about the reciprocal relationship between context (spatial organization, environment, etc.) and individual behavior. This emerging research community has yet to adequately address the new threats to the confidentiality of respondent data in spatially explicit social survey or census data files, however. This paper presents four sometimes conflicting principles for the conduct of ethical and high-quality science using such data: protection of confidentiality, the social-spatial linkage, data sharing, and data preservation.

108. Onsrud, H.J. "**Identifying Unethical Conduct in the Use of GIS,"** Cartography and Geographic Information Systems, 1995, 22(1), 90-97 http://www.spatial.maine.edu/~onsrud/pubs/ethics18.pdf

Describes and discusses various "grey areas" in the use of GIS and in determining what constitutes a beneficial versus a detrimental consequence, and how these often depend on the perspectives of those affected by the use of information systems.

Electronic security

109. International Telecommunication Union **"Security in Telecommunications and Information Technology An Overview of Issues and the Recommendations for Secure ITU-T,"** 2012. (To purchase this article please use the following link: http://www.itu.int/pub/T-HDB-SEC.05-2011)

This manual provides a broad introduction to the ICT security work of the ITU-T and, more specifically, it summarizes how the ITU-T is responding to global cybersecurity challenges with Recommendations, guidance documents and outreach initiatives. It is primarily directed towards those who have responsibility for, or an interest in, information and communications security and the related standards, as well as those who simply need to gain a better understanding of ICT security issues. The manual can be used in various ways according to the organization, role and needs of the user. The introductory chapters provide an overview of the current key areas of the ITU-T security work together with a discussion of the basic

requirements for the protection of ICT applications, services and information. The threats and vulnerabilities that drive security requirements are highlighted and the role of standards in meeting the requirements is examined. Some of the features that are needed to protect the various entities involved in providing, supporting and using information and communications technology and services are discussed. In addition, the importance of ICT security standards is explained and examples are given of how the ITU-T security work is evolving to meet security requirements.

110. Morse RE, Nadkarni P, Schoenfeld DA, Finkelstein DM. **"Web-browser encryption of personal health information,"** *BMC Med Inform Decis Mak*. 2011;11:70. doi:10.1186/1472-6947-11-70. http://www.biomedcentral.com/1472-6947/11/70

Electronic health records provide access to an unprecedented amount of clinical data for research that can accelerate the development of effective medical practices. However it is important to protect patient confidentiality, as many medical conditions are stigmatized and disclosure could result in personal and/or financial loss. This publication describes a system for remote data entry that allows the data that would identify the patient to be encrypted in the web browser of the person entering the data. These data cannot be decrypted on the server by the staff at the data center but can be decrypted by the person entering the data or their delegate. We developed this system to solve a problem that arose in the context of clinical research, but it is applicable in a range of situations where sensitive information is stored and updated in a database and it is necessary to ensure that it cannot be viewed by any except those intentionally given access.

111. Oracle Corporation **"Security and Compliance with Oracle Database 12 C"** 2014;(April). http://www.oracle.com/technetwork/database/security/security-compliance-wp-12c-1896112.pdf

Includes sections on authentication, authorization, access control, identity management, encryption, monitoring, and accountability. The document shows that this version of Oracle is "privacy oriented" and compatible with our guidelines: authentication, role-base access, encryption (AES…), notion of Virtual Private Databases, etc. Concludes with a chapter on the challenges of technology to data protection

112. Richard E. Smith "**Internet Cryptography,"** 1997, Addison-Wesley, ISBN**:** 0201924803

Describes the mathematics behind computer encryption schemas, as well as the overall strategy behind public-private and other strategies for authorized decryption of information.

113. Sidnie Feit, McGraw-Hill "**TCP/IP: Architecture, Protocol, and Implementation with IPv6 and IP Security,"** 1999 McGraw-Hill Computer Communications Series ISBN**:** 0070213895

Additional information on securing information systems which are deployed over the internet. This book is also intended for technical audiences.

114. Stephen A. Thomas "**SSL and TLS Essentials: Securing the Web,"** 2000 John Wiley and Sons, ISBN**:** 0471383546

A book-length, technical explanation of how secure sockets layer (SSL) encryption works.

115. US DHHS/ National Committee on Vital and Health Statistics "**Cryptography-based Patient Identifier,"** 1997 http://ncvhs.hhs.gov/app7-4.htm

A short article on the role of encryption in electronic security.

116. Uyless D. Black "**Internet Security Protocols: Protecting IP Traffic,"** 2000
Prentice Hall PTR; ISBN**:** 0130142492

A book-length, technical explorations of the issues associated with security internet traffic.

Web links

CDC and HIV/AIDS: http://www.cdc.gov/hiv/

UNAIDS: http://www.unaids.org/en/

USAID and HIV/AIDS: http://www.usaid.gov/our_work/global_health/aids/

WHO and HIV/AIDS: http://www.who.int/hiv/en/

UK Department of Health: http://www.dh.gov.uk
